# Self-Transcendent Emotions and Their Social Effects: Awe, Elevation and Kama Muta Promote a Human Identification and Motivations to Help Others

**DOI:** 10.3389/fpsyg.2021.709859

**Published:** 2021-09-13

**Authors:** José J. Pizarro, Nekane Basabe, Itziar Fernández, Pilar Carrera, Pedro Apodaca, Carlos I. Man Ging, Olaia Cusi, Darío Páez

**Affiliations:** ^1^Department of Social Psychology, University of the Basque Country, San Sebastian, Spain; ^2^Department of Social Psychology and Organizations, The National Distance Education University, Madrid, Spain; ^3^Department of Social Psychology and Methodology, Autonomous University of Madrid, Madrid, Spain; ^4^Department of Research and Diagnostic Methods, University of the Basque Country, San Sebastian, Spain; ^5^Faculty of Philosophical-Theological Sciences, Pontifical Catholic University of Ecuador, Quito, Ecuador

**Keywords:** self-transcendent emotions, human identification, collective action, awe, elevation, Kama Muta

## Abstract

Abundant literature shows the effects of negative emotions on motivations to engage in collective action (i.e., to collectively mobilize personal resources to achieve a common objective), as well as their influence on the creation of shared identities. In this proposal, we focus on the possible role of Self-Transcendent Emotions (STEs) defined as positive-valence emotions that have been key in the creation and maintenance of collective identities, as well as in promoting individuals well-being. In detail, we examine their influence in (a) strengthening a global identification, (b) increasing willingness to collectively help others, and (c) improving people’s wellbeing. For this reason, we conducted a preliminary literature review of *k* = 65 independent studies on the effects of STEs on connection to others. Through this review (fully available in Supplementary Materials), we selected a sample of STEs (Awe, Elevation, and Kama Muta) and elicitors to conduct a video-base study. In it, 1,064 university students from 3 different cultural regions (from Spain and Ecuador) were randomized to answer one of three STE scales (i.e., each measuring one of the selected STEs), and evaluate three videos in random order (i.e., each prototypical for the selected STEs). Participants also answered a measure of global identification and intentions to collectively help others (after each video), as well as self-transcendent and well-being (at the end of the survey). Results from SEM analyses show these STEs motivated a fusion of identity with all humanity, as well as collective intentions to help others, even controlling for individuals’ value orientations. In addition, the three of them indirectly increased participants’ well-being through a higher global identity. While there are differences among them, these three STEs share common elements and their effects are constant across the different cultural regions. It is concluded that Awe, Elevation, and Kama Muta, even individually experienced, have a significant potential to influence people’s behavior. Specifically, in various forms of collective action aimed at helping others.

## Introduction

Why do some people engage in prosocial behaviors toward strangers? Why would I feel motivated to participate and help others? Questions like these have deeply intrigued thinkers of social behavior, and the topic of helping others, especially non-kin, has been considered an “altruism puzzle”; particularly, when trying to explain the factors that have influenced these behaviors to evolve (e.g., Hamilton, 1964; Fehr and Fischbacher, 2003; Van Vugt and Van Lange, 2006; Jensen et al., 2014). From a perspective centered on collective behaviors, prosociality toward others (i.e., to mobilize personal resources to help non-ingroup members such as the underprivileged, social minorities, etc.) has been largely studied under the form of different forms of collective action (i.e., collectively behave with a common objective). For this reason, there is currently a great amount of theoretical and empirical works oriented at analyzing its antecedents, processes, and consequences (Klandermans and Roggeband, 2010).

Regarding the factors that create, shape, and result from these motivations and behaviors, different studies can be found analyzing beliefs of group efficacy (Klandermans, 1984; Bandura, 2000), collective identities (Drury and Reicher, 2005; Neville and Reicher, 2011), and emotions (Jasper, 1998; van Troost et al., 2013) (to review comprehensive models, see van Zomeren et al., 2008; also, Thomas et al., 2009). Nevertheless, when centering the attention on the role of the emotions at play, more often than not negative emotions have been analyzed (e.g., anger), while positive ones (e.g., love), have receive less attention. Rather, they have been usually addressed as possible mediators or mechanisms that arise in collective participation (Jasper, 1998; van Troost et al., 2013).

Here, an alternative proposal is presented, focused on the role of positive emotional experiences that can ultimately encourage uninterested help and concern toward others, based on different theories about Self-Transcendence and the role of Self-Transcendent Emotions (see Stellar et al., 2017b). In specific, how the latter –experienced in individual settings– is able to orientate the individual self to concerns and the welfare of others and motivates collective action to promote greater good and a common social identification.

## Emotions and Solidarity

In the study of emotions, there is a great consensus on the functionality they have in human life (Keltner and Haidt, 1999; Fischer and Manstead, 2008; Niedenthal and Brauer, 2012; Lench, 2018). These functions help propitiate automatic and adaptive responses with the ultimate goal of increasing human survival. Since the pioneer works of Willian James and Charles Darwin, the study of emotions has varied considerably (for a historical perspective, see Gendron and Barrett, 2009). Nevertheless, it is possible to conceptualize them as automatic and brief affective reactions that unchain a multi-componential response (e.g., physiological changes, action tendencies, affective responses) which includes diverse human systems (Frijda, 2000; Scherer, 2005, 2009; Moors et al., 2013). In relation to the specific functions that diverse emotions have, on the other hand, it is possible to find various lines of research. For example, some researchers have studied their role as moral amplifiers (Haidt, 2003b; Tangney et al., 2007; Horberg et al., 2011), regulators of social relationships (Fiske, 2002; Gervais and Fessler, 2017), and more related to group-related social relationships, as accelerators or amplifiers of human behavior (van Stekelenburg and Klandermans, 2007).

In this particular setting centered on prosociality and collective action, appraisal theories gain greater relevance (van Troost et al., 2013). One of the differential aspects of these theories is the person- environment relationship and the appraise of the relevance of different events (i.e., elicitors) for well-being (for a review, see Moors et al., 2013). In this manner, it is possible to explain, for instance, why some but not everyone can feel admiration toward the figure of a political martyr or anger due to the lack of political and instrumental efforts regarding the climate crisis. Lastly, these perspectives in the study of emotions are still of great significance due to the broad scope of effects on people’s lives. This is because virtually every emotional experience –including those lived individually– has an impact in subsequent social interactions (Rimé, 2007, 2009). Therefore, to better understand collective behaviors both naturally occurring (e.g., collective action and social movements), as well as those instrumentally produced (e.g., campaigns, governmental, and non-governmental initiatives), the study of individual and collective experiences of emotions are indispensable.

### Negative Emotions and Collective Action

In different attempts that seek to explain how emotions can be related to the pursuit of collective gains (i.e., over individuals’), several researchers tend to point to the importance of punishment-based approaches as the proximal mechanisms in the evolution of particularly non-kin altruism (e.g., Yamagishi, 1986; Fehr and Gächter, 2002; Boyd et al., 2010). Thus, it is the negative affect (e.g., avoidance of punishment in the form of fear) that can serve as a motivator, but also, as a disruptor of coordinated efforts that maximize gain in a dyadic task (Kuroda and Kameda, 2019).

Analyzing how negative emotions can affect, on the other side, collective behaviors, a great deal of attention has been given to the role of anger (for a review, see van Troost et al., 2013). For instance, it has been shown its effects on signing a petition denouncing abuse and mistreatment (Miller et al., 2009), promoting demonstrations and hunger strikes at the beginning of the 1989 Chinese Student Movement (Yang, 2000), in the collective participation afterward the suicide protest in South Korea in 1991 (Kim, 2002), and supporting intentions of reparations and compensations in the context of United States and British occupation in Iraq (Iyer et al., 2007). Another negative emotion that has received attention in the context of outgroup solidarity is guilt (for an operationalization, see Tangney et al., 2007); in particular, collective guilt, which depends on the identification a person has to a specific social group (Doosje et al., 1998). For example, this emotion has been shown as an important predictor of willingness to repair the past wrongdoings of one’s particular group in different settings, such as the Mapuche conflict in Chile (Brown et al., 2008), or regarding the role of one’s country in the current climate crisis (Ferguson and Branscombe, 2010).

Other forms of how negative emotions’ effect on collective action are those presented in theories based on morality and people’s ethical principles. In these, negative affect has a predominant role even though not always explicitly stated. To illustrate, diverse studies highlight the intense and persistent emotional responses felt in cases of perceptions of injustice experienced by underprivileged people (i.e., moral outrage) (Hoffman, 1987; Montada and Schneider, 1989) –see also Jasper (2010); as a pure form of anger, see the work of Batson et al. (2007). Another example is through a motivational principle based on one’s own morality which, in case of not being satisfied, could entail a high personal cost (i.e., moral obligation) (Sabucedo et al., 2018). As these research lines implicitly suggest, collective participation could also be conceptualized in function of avoiding or minimizing negative emotions (i.e., in the case of moral outrage) or preventing its future appearance (i.e., moral obligation).

In all, these approaches go in line with different emotion reviews, such as that of Haidt (2003b) or Keltner and Lerner (2010) who show how different emotions contribute –among other functions– to align one’s attention with moral concerns such as human rights, justice, and benevolence.

### Positive Emotions and Collective Action

The case of the study of positive emotions, conversely, has not received considerable attention as one might expect and has even yielded contradictory results. For instance, two emotions that might be intuitively associated to promote collective action –due to their cognitive and motivational effects– are compassion for others (Goetz et al., 2010; Strauss et al., 2016) and hope (Snyder, 2002; McGeer, 2004). Despite these emotions can motivate to help other’s suffering and promote agency thinking to achieve desirable goals (respectively), there are not many studies that analyze their role in promoting collective action –for a notable exception, see Pagano and Huo (2007).

In studies focusing on the role of hope and collective action engagement, for example, this emotion has been proved to be a facilitator of collective action but only when is high (Cohen-Chen and Van Zomeren, 2018; see also Westoby and McNamara, 2019), or as a sequential prerequisite to canalize anger, which in turn increases the willingness to collectively participate (Wlodarczyk et al., 2017). At the same time, the coping functions of hope could back-fire and even decrease helping intention and resources mobilization in the context of the current climate crisis (van Zomeren et al., 2019).

Overall, the role of emotions is undisputable in the study of collectively helping others and yet, greater attention has been constantly attributed to the role of negative ones. In the following section, different theorizations of Self-Transcendence are presented with the intention to introduce Self-Transcendent Emotions (respectively, ST and STEs, hereafter) which are proposed as a taxonomy of positive emotions that can motivate human behaviors toward the needs and promotion of welfare of others and thus, have important implications in collective action.

## Self-Transcendence

In the theorization of ST, we can find Maslow’s (1964) peak experiences (see also Koltko-Rivera, 2006) as a generalized term to refer to situations where people can reach a highly positive state of self-transcendence and fulfillment that includes different spheres (e.g., social, religious, political, etc.) (see also Maslow, 1962). Through these instances, the individual transcends him or herself with a sense of goodness, benevolence. In addition, they connect with everything that surrounds themselves and this experience is considered to be inherent to all human beings (Frankl, 1966).

Contrary to this momentary-experience approach, there is also the position that explains ST as a process that receives the boost of different trait-type factors in order to take place. This description of ST goes in line with what Schwartz’ (1994) theorized as a group of universal value and motivational orientations. After reviewing different models of personal values (e.g., Rokeach, 1973), and how they should be ordered (e.g., Schwartz and Bilsky, 1990), Schwartz (1994) proposed an aggrupation of different human motivations, where hierarchies and affinities are stablished. In the particular case of ST, the author posits that it is an orientation that guides individuals to ideals of universality (e.g., justice for everyone) and benevolence (e.g., help and loyalty to close ones). This dimension is orthogonal to those that imply self-enhancement, as for instance, achievement values (i.e., “Personal success through demonstrating competence according to social standards,” pp. 22), and thus, there is a continuum that inexorably depends on the degree of involvement to one’s self (i.e., from enhancing ourselves to enhancing others). Connected to prosociality, several studies have confirmed their role in enhancing different forms of prosocial behaviors (e.g., Oceja and Salgado, 2013; Daniel et al., 2015; Bayram, 2016).

In all, both approaches share the assumption that the individual self that is somehow outwards oriented (e.g., other peoples, the environment, etc.), and, as Yaden et al. (2017) argue, this orientation can take several forms. According to these authors, several experiences and psychological processes allow people to live ST-related states. Indeed, they focus on temporary states that can –even briefly– produce a reduction in the self-oriented goals and a greater sense of connection with what surrounds us. In their revision, they list how different instances of – for instance – Mindfulness, Flow, and Mystical experiences meet these criteria and further, as well as STEs.

### Self-Transcendent Emotions

#### Attributes and Functions of STEs

Self-transcendent emotions can be defined as a classification of human emotions that orientate our personal selves toward outside (e.g., other peoples’ needs) and thus generate a change from our self-absorption, concerns, and selfish goals (Fredrickson, 2013; Van Cappellen and Rimé, 2014; Stellar et al., 2017b). These emotions are thought to play a major role in the manifestation of spiritual and religious practices, as the search of meaning (Van Cappellen, 2017; see also Emmons, 2005).

The study of these emotions has received greater interest quite recently, and their most differentiating characteristics are described in terms of two central attributes. Namely, (i) they should be mainly elicited by stimuli that are not completely directed to the individual self (Haidt, 2003a; Van Cappellen and Rimé, 2014; Stellar et al., 2017b); and (ii), they promote a connection or union with other people and groups. The latter could be manifested –for instance– in terms of increased prosocial behavior tendencies (Haidt, 2003b; Stellar et al., 2017b), care-taking behavior of others (Van Cappellen and Rimé, 2014), or a socio-emotional identification with highly inclusive groups which can be sustained by collective participation in rituals (de Rivera and Carson, 2015; de Rivera, 2018).

Under these criteria, some emotions that have been considered part of this taxonomy are studied under the name of Awe (Keltner and Haidt, 2003), Elevation (Haidt, 2003a; for a review, see Pohling and Diessner, 2016), Gratitude (for a meta-analytical review, see Ma et al., 2017), Kama Muta (in Sanskrit, being moved by love; Fiske et al., 2016; see also Zickfeld et al., 2018), Compassion (for a review, see Goetz et al., 2010; see also Strauss et al., 2016), or even Admiration (Onu et al., 2016; see also, Schindler et al., 2013).

When analyzing the ultimate reason of why these emotions occur, diverse authors agree on the fact that they boost a sense of connection with other people (e.g., Van Cappellen and Rimé, 2014; Stellar et al., 2017). This way, experiencing a STE promotes group-forming and commitment-maintenance processes with specific objectives, and consequently, one’s survival probability is higher. In this line, most of STE-models and explanations rely –implicitly or explicitly– on the notion that they should have been affected by different phenomena at play during the evolution of our psychology. One of these, for example, has been cultural evolution, which has affected the way in which human beings create and maintain identities with kin and non-kin (see Henrich, 2020; also Atran and Norenzayan, 2004; Richerson et al., 2016). Due to the fact that human beings are born with highly adaptive skills for social life (Henrich and Gil-White, 2001; Herrmann et al., 2007; Thomsen et al., 2011), it is natural to think that some emotions could motivate –while not being restrictive to them– behavioral patterns oriented to maximizing future instances of social life and group cohesion, specially, when the groups are at early stages of constitution. Empirically, we can group a large body of evidence that provides support for this idea, being many of those, different forms of prosocial behavior.

### Empirical Research on STEs

As a preliminary attempt at examining the nature and the effects of a variety of STEs, we conducted a non-systematic review of studies. For this, we selected studies measuring Awe, Elevation, Gratitude, Compassion (to others), and Kama Muta, to analyze (a) their elicitors and (b) their effects on promoting inter-personal and inter-group connections (for the full review with *k* = 65 independent studies, see Supplementary Materials Online^1^). We selected these particular STEs for two reasons. The first, because these emotions fulfill the criteria of shifting the attention away from one’s self-absorption and needs, and to promote (i.e., indirectly or directly) people to join and unite in larger groups (Stellar et al., 2017b). The second, because we wanted to analyze the stimuli used in experiments and in field studies. In this way, a more accurate selection of stimuli could be generated for the present study.

Table 1 summarizes empirical findings of the included studies (for the full table, see Supplementary Materials Online). As one can observe, virtually all of them are being elicited by stimuli that make people place their attention outside themselves; in other words, to put one’s immediate needs aside. These examples include the attempts of using the nature and space (i.e., in the case of Awe), people helping others or recognizing their importance (i.e., Elevation), or the expression of thankfulness and appreciation to others (i.e., Gratitude).

**TABLE 1:** Review (summarized) of the effects of STEs on connection with others.

Emotion	References	Study, elicitors or measurement of interest	Effects on connection to others	Effect on behavior	
				Direct	Indirect
Awe	Piff et al., 2015	S2: recalling event. S3: video of nature (from BBC’s *Planet Earth*). S4: video of threatening phenomena, and colored droplets in slow motion (from *The Slow Mo Guys*).	An increase of ethical thinking and helping behavior (S2), more generosity in a dictator game (S3), and increased prosociality for resource allocation (in both awe conditions in S4).	X	X
Elevation	Aquino et al., 2011	S2: recalling event. S3: priming of moral identity and reading news of forgiveness. S4: video of donation to several charities (*World on Fire*).	S2: elevation emotions were associated to a greater motivation to help others. S3: greater prosocial behavior (modified dictator game).	X	X
Gratitude	Williams and Bartlett, 2014	Receiving a note expressing gratitude in a mentoring program.	Perceived writers as more appreciative, warmer, higher affiliative intentions, and more people leaving contact information.	X	X
Compassion	Lim and DeSteno, 2016	S1: self-reported measure of dispositional compassion (correlational). S2: observing an ill person completing a tedious task.	Greater intentions of donations to a charity (S1), and more time helping a person with a tedious task (S2).	X	X
Kama Muta	Seibt et al., 2018	S1 and S2: videos of emotionally moving political campaigns.	Greater intention to support the political candidate (S1 and S2).		X

*The number of the particular study where each stimulus was used is referenced after the S. Effects on behavior are classified in terms of direct or indirect. That is, whether the main effects described would impact it directly (actual behavior, such as amount of time devoted to help) or indirectly (tendency or motivation to, such as increases intention to help others), respectively. The full table (k = 65 studies) can be seen in Supplementary Materials online.*

Additionally, Table 1 and Supplementary Table 1 shows an evaluation on whether the impact of these results in connection with others. Specifically, these outcomes are classified in terms of producing direct effect on peoples’ behaviors that facilitates integration/solidarity/union or commitment to others (i.e., carrying out a behavior that promotes future interactions at dyad- or bigger levels), indirect effects (i.e., preparing a disposition or motivation that might end up in further interactions at dyad- or bigger levels), or both. In this manner, we can see (for instance) a greater display of caring behaviors, and more altruistic distribution of resources (i.e., direct effects) (Tsang, 2006; Silvers and Haidt, 2008, for Elevation and Gratitude, respectively). Also, more self-reported perceptions of feeling small and connected to something bigger, and humility (i.e., indirect effects) (Shiota et al., 2007; Stellar et al., 2017a, for Awe). Finally, there are also those studies where the authors provide both direct and indirect evidence. For instance, the case of Schnall et al. (2010) study, where the authors report both a greater intention to morally improve and, in addition, more time dedicated to help the experimenter as a result of evoking Elevation.

In these studies, we can see not only how these emotions have been examined in experiments and in other kind of studies, but also that they implicitly tap a core tenet of human life: creation and maintaining of social identities. The fact that different theorization of these emotions includes this as a fundamental characteristic, along with the effects derived from this review, suggests that they play –and have played– an important role in the development of groups (for an analysis of awe in rituals and growing complexity of societies, see Henrich, 2020).

## STEs, Inclusive Identities, and Collective Action

A great deal of empirical studies suggests that these emotions can have an important role in predicting a collective form of prosocial behavior toward others, as well as to motivate a greater sense of identification with others. In this regard, an approximation on the effects of shared identities should be done, with emphasis on how they can contribute shaping different forms of collective action.

In the core of different forms of collective behaviors, there are the social identities. Based on the Social Identity Theory (Tajfel and Turner, 1979), Turner et al. (1987) developed the notion of different levels of inclusiveness in the process of individual self-categorization. This approach (i.e., the Social Identity Approach, see Hornsey, 2008) has been taken into account in theoretical and empirical studies on collective action (for a review of identities and collective action, see van Stekelenburg and Klandermans, 2007) and it continues to influence different models of this form of prosocial behavior. The influence of shared identities in different forms of collective participation has been studied as prerequisites (Thomas et al., 2009), outcomes (Páez et al., 2015) or both (Drury and Reicher, 2005), and as moderators of several relationships in the literature (e.g., Shepherd et al., 2013; Krauth-Gruber and Bonnot, 2019).

In the context of a highly inclusive human identification (Turner et al., 1987), studies consistently show that this form of self-categorization is associated to different forms of prosocial behaviors (see McFarland et al., 2013, 2019; Buchan et al., 2017), where both the helper and the *helpee* could be the members of the same group. Therefore, the study of ST related to superordinate identities raises a question about the nature of the motivation to engage in collective action toward the common good: Is a highly shared identity a fundamental pre-requisite for helping others, or can it be an outcome of STEs?

With this question, it would be possible to integrate a part of highly extended models of collective action that include –many times as a necessary condition– a shared identify (van Zomeren et al., 2008). Nevertheless, the context of superordinate identities does not necessarily correspond to the contingencies of classic literature of collective action. Specifically, because this context is not usually that of ingroups and outgroups (e.g., Klandermans et al., 2002; Drury and Reicher, 2005; Neville and Reicher, 2011), but a highly inclusive one that might not be frame the way different social identities are (see Roccas and Brewer, 2002). Therefore, while a higher degree of human identification is indeed a precursor of prosocial behaviors to others, and at the same time, STEs are able to enhance it, there is not sufficient evidence to stablish specific hypotheses suggesting that this identification is a pre-requisite to influence the effects of STEs on prosociality.

## Objectives and Hypotheses

The main objectives of this study are twofold: it is aimed at analyzing the pattern of STEs responding to different stimuli, and then, the psychosocial outcomes they produce. Specifically, we want to evaluate Awe, Elevation and Kama Muta responses to prototypical elicitors used in previous studies and subsequently, to evaluate their predictive power on the willingness to engage in different collective action forms and on a superordinate category of identity (i.e., fusion of identity with everyone in the world).

Considering the frameworks these emotions have been theorized from (i.e., Haidt, 2000; Keltner and Haidt, 2003; Fiske et al., 2017a, respectively), and different attempts at distinguishing them (e.g., Zickfeld et al., 2018; Pizarro et al., 2021), we expect that emotional responses measured with specific scales will be most intense and result in a distinguishing pattern when each emotion is provoked by its prototypical elicitor (see review of studies in Supplementary Materials). In other words, the vastness of nature, a virtuous and moral example, and an intensification of a communal sharing relationship will provoke the most intense reactions for Awe, Elevation and Kama Muta, respectively (for this proposed classification, see Onu et al., 2016). However, an opposite trend can be expected, at the same time. Here, that some scales (particularly those of Awe and Elevation) are also able to capture the apparition of this emotion in contexts involving social content, such as a benevolent leaders (i.e., prototypical for Elevation) or even a show of deep gratitude (i.e., prototypical for Kama Muta) (see for example, Zickfeld et al., 2017). For this reason, for H1, we have competing hypotheses: on the one side, we can expect that these three emotions are indeed differentiable through stimuli and scale; conversely, that these emotions co-occur and vary across stimuli (i.e., H1 and H1’, respectively). In each case, we do expect that in all we can see a pattern of Self-Transcendence.

Based on previous studies (e.g., Cusi et al., 2018; Pizarro et al., 2018; Pizarro, 2019), we predict that each of these emotions will strengthen the human-level of identification (H2), and will provoke intentions on engaging in collective behaviors to help other peoples (H3). For these hypotheses, we also predict that the emotional effects will continue even controlling for individual orientations and differences which have been proved to relate to helping behavior (see Oceja and Salgado, 2013; Daniel et al., 2015; Bayram, 2016), as well as identifications with all humanity (e.g., McFarland et al., 2019). Finally, we also wanted to test whether these emotions can indeed produce –as side effects– any impact on people’s wellbeing (H4), as it has been found before. This can be produced by the emotions themselves (e.g., Amutio et al., 2018; Cusi et al., 2018; Pizarro et al., 2018) or through the possible performance of act of kindness to others (e.g., Curry et al., 2018).

## Materials and Methods

### Participants and Procedure

1,063 university students from 3 universities participated in the study (53.5% women) with ages from 18 to 69 (*M* = 32.13, *SD* = 12.12); according to the university:

• UPV (University of the Basque Country, Spain), *n* = 112, 74.1% women, aged 18–40 (*M* = 20.19, *SD* = 3.48).
• PUCE (Pontifical Catholic University of Ecuador), *n* = 256, 52% women, aged 18–62 (*M* = 21.36, *SD* = 4.29).
• UNED (National Distance Education University, Spain), *n* = 695, 50.8% women, aged 18–69 (*M* = 37.63, *SD* = 10.99).

They were randomly assigned to one of three conditions, according to the emotional scale they would have to complete^2^ : Awe (*n* = 359; 56.8% women; aged 18–60, *M* = 31.84, *SD* = 11.79), Elevation (*n* = 338; 51.8% women, aged 18–69, *M* = 32.65, *SD* = 12.49), and Kama Muta (*n* = 366; 51.9% women, aged 18–67, *M* = 31.92, *SD* = 12.18). In each condition (which could be thought as independent intra-subject studies), participants watched three videos aimed at eliciting Awe, Elevation and Kama Muta (also in a random order), based on a prototypical stimulus, and, after watching each one, they completed the scale assigned to their condition (see Table 2). The selection of stimuli was in concordance with empirical studies published under the name of each emotion, and based on the review of studies. In the case of Awe and Elevation, the reviews took into account Keltner and Haidt’s (2003), and Haidt’s (Haidt, 2000, 2003a; Algoe and Haidt, 2009) (respectively) theoretical conceptualizations, as well as a series of studies based on free-recalling previous experiences of both emotions (Cusi et al., 2018; Pizarro et al., 2018, respectively). For the case of Kama Muta, the stimulus was selected based on a prototypical intensification of a Communal Sharing (CS, hereafter) form of relationship (Fiske, 1991, 1992), which had been already used in a cross-cultural study aimed at measuring Kama Muta (Zickfeld et al., 2018).

**TABLE 2:** Overview of the videos and scales used in the congruent conditions.

	Videos and scales		
	Awe Mongolian Horse Riders (*n* = 359)	Elevation The story of Mandela (*n* = 338)	Kama Muta The generosity of a Thai Dr. (*n* = 366)
Characteristics of the video	Focused on the nature, aerial shots of horse-riders in Mongolian meadows, from the documentary. Final shot is zoom out to increase vastness. Little or non-existing social interactions (3:29 length).	Story of Nelson Mandela’s life and greatest achievements. It is an animated series of pictures with descriptive text focusing on his exemplary life. Little or non-existing social interactions (4:42 length.	Intensification of a communal sharing relationship. A Thai man’s doctor cancels his patient’s medical bill because of the gratitude he had showed him years before (3:02 length).
Scales	Awe scale: multi-component experience (16 items, 1–7 scale)^1^ (Pizarro et al., 2018).	Elevation scale: multi-component experience (19 items, 1–7 scale) (Cusi et al., 2018).	KAMMUS: multi-component experience (23 items, 0–6 scale) (Zickfeld et al., 2018).
Scale description	Appraisals (2; separately positive and negative), affective response/labels (3), physiological response (3), cognitive-subjective response (4), action tendency (3).	Appraisals (2), affective response/labels (5), physiological response (4), cognitive-subjective response (4), action tendency (4).	Tears (2), chills (2), warmth (2), speaking difficulties (3), enthusiasm (3), appraisals (4), motivations (4), Eeotion labels (3).
Example of distinguishing elements	Affective response/labels: *admiration*, *wonder*, *in-awe*, *amazed*.	Affective response/labels: *inspired*, *elevated*, *enthusiastic*, *illuminated*.	Labels: *heartwarming*, *moved*, *touched*.

**FIGURE 1:**
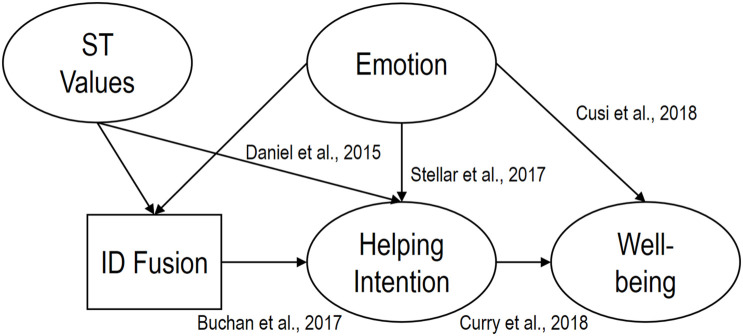
Theoretical model of the effects of STEs. It represents the relationships tested in the present study through SEM analyses. Circles represent the relation measured by 2 or more items, while the square (identity fusion with all humanity), a single pictorial item. The model includes references to theoretical and/or empirical studies showing the relationships. ST Values, Self-Transcendent Values; ID Fusion, Identity Fusion. These models (i.e., one for each STE) as well as complete figures (with standardized coefficients), can be seen in Supplementary Materials Online.

*To view the videos, anyone can write directly to the correspondence author. ^1^Due to statistical results the present study used a version without the appraisals. However, both appraisals’ intensities can be seen in Figure 1.*

After each video and the emotional scale, participants answered items that measure their Helping Intention to others and a pictorial item that measure their Identity Fusion with all humanity. Finally, after concluding the three videos and measures, participants filled the final section, which included Transcendent Values, Well-being, and general demographic information. The application was conducted online (Qualtrics), in Spanish, and took about 35 min to be completed.

### Instruments

Awe scale (Pizarro et al., 2018). 16 items were used that evaluate an awe-eliciting experience based on Keltner and Haidt’s (2003) definition and a free-recall of past event, from a multi-faceted orientation. The scale includes Appraisals (e.g., *I feel in the presence of something grand*), Affective response/labels (e.g., *I’m in awe before something grand*), Physiologic responses (e.g., *I feel the shivers*), Cognitive-subjective response (e.g., *I feel small*), and Action tendencies (e.g., *I wish to be part of something bigger that myself*). α = 0.946, and ω = 0.954 (Affective response/labels), α = 0.871, and ω = 0.867 (Physiologic response), α = 0.859, and ω = 0.885 (Cognitive-subjective response), and α = 0.863, and ω = 0.865 (Action tendencies); the total of the scale was α = 0.950, and ω = 0.932 (see Supplementary Materials).

Elevation scale (Cusi et al., 2018). Participants answered 19 items that evaluate the experience of elevation toward great exemplars of morality (Haidt, 2003a) and a free-recall of past event, through a multi-faceted scale. The scale includes Appraisals (e.g., *I’m in the presence of an exceptionally kind and moral person*), Affective response/labels (e.g., *I feel inspired, elevated by him/her*), Physiologic responses (e.g., *I feel a nice and warm sensation in the stomach*), Cognitive-subjective response (e.g., *I feel optimistic after witnessing a virtuous person*), and Action tendencies (e.g., *I wish to be a better person after witnessing this example*). α = 0.920, and ω = 0.923 (Appraisals), α = 0.949, and ω = 0.952 (Affective response/labels), α = 0.905, and ω = 0.913 (Physiologic response), α = 0.903, and ω = 0.911 (Cognitive-subjective response), and α = 0.959, and ω = 0.961 (Action tendencies); the total of the scale was α = 0.964, and ω = 0.969.

Kama Muta Multiplex Scale - KAMMUS (Zickfeld et al., 2018). This scale included 23 items oriented at measuring an emotional response to intensification of communal sharing relationships. Participants had to indicate the extent they felt different sensations in the dimensions of Tears (e.g., *Moist eyes*), Chills (e.g., *Chills or shivers*), Warmth (e.g., *A warm feeling in the center of the chest*), Speaking difficulties (e.g., *A lump in the throat*), Enthusiasm (e.g., *Refreshed, energized, or exhilarated*), Appraisals (e.g., *A unique kind of love spring up*), Motivations (e.g., *I felt like telling someone how much I care about them*), and Labels (e.g., *I was moved*). α = 0.867, and ω = 0.871 (Tears), α = 0.888, and ω = 0.888 (Chills), α = 0.877, and ω = 0.877 (Warmth), α = 0.828, and ω = 0.834 (Speaking difficulties), α = 0.616, and ω = 0.638 (Enthusiasm), α = 0.914, and ω = 0.915 (Appraisals), α = 0.912, and ω = 0.914 (Motivations), and α = 0.836, and ω = 0.866 (Labels). The total reliability of the scale was of α = 0.947, and ω = 0.955.

Transcendence values (Schwartz, 2007). 5 items were used representing the dimension of self-transcendence values. Each participant had to indicate how much he or she felt they looked like a person (e.g., She thinks it is important that every person in the world be treated equally. She believes everyone should have equal opportunities in life) in a scale from 1 (Not at all like me) to 6 (Very much like me). α = 0.751, and ω = 0.759.

Help Intention in different NGOs (*ad hoc* items). Participants indicated their agreement with 4 items that expressed the intention to carry out different collective actions oriented at helping others or the nature (e.g., … participating in collective demonstrations [e.g., street demonstrations] to support humanitarian issues? or … collaborating with an NGO regarding humanitarian help?), in a scale from 1 (Not at all) to 5 (Very much). Reliability indexes for the congruent stimulus-scale were α = 0.817, and ω = 0.823 (Awe condition); α = 0.813, and ω = 0.821 (Elevation condition); α = 0.831, and ω = 0.837 (Kama Muta condition).

Identity Fusion (adapted from Swann et al., 2009). A pictographic single-item measure was used to assess identity fusion with people in general. The main instruction was: “Please select the drawing that best describes your relationship with people in general.” This picture is represented in a 5 point Likert scale where the highest point represents the total inclusion of the personal self in the highest-level group (i.e., everyone in the world).

Well-being (Hervás and Vázquez, 2013). Finally, we measured participants’ well-being through two items (I am very satisfied with my life; I have the energy to accomplish my daily tasks) on a 0 (totally disagree) to 10 scale (totally agree). These items are aimed at measuring a general aspect related to global satisfaction with life and eudaimonic functioning.

### Data Analyses

All analyses were conducted in R with RStudio (RStudio Team, 2015). First, we explored the factorial structures of all variables used; we carried out several CFA analyses with the *lavaan* package (Rosseel, 2014) to evaluate the factorial structure of the scales (using only the congruence video-scale), and then, we analyzed the intensities of the responses to every video from each condition, with the package *ggplot2* (Wickman, 2016) (for H1 and H1′).

Subsequently (for H2–H4), we explored the effects each stimulus with the *a priori* selected scale (i.e., congruence video-scale). Thus, we explored the impact of the variables through correlational analyses (using *apaTables* package; Stanley, 2018) and subsequently, conducted several random-model meta-analysis of the main variables for each condition with the *metaphor* package (Viechtbauer, 2015). These analyses provided a better-estimated effect sized of each relationship in the 3 universities to evaluate possible sources of heterogeneity for the three regions (i.e., the Basque Country, Ecuador, and Spain). Finally, we explored all the theoretical relationships (see Figure 1) through 3 SEM analyses. To establish the criteria for interpretation of the main analyses (i.e., SEM), we reviewed previous literature published with these emotions (e.g., Zickfeld et al., 2018). Therefore, we considered the SEM models to be adequate to interpretation when they the value of CFI is above 0.95, RMSEA lower than 0.10, and lower than 0.08 for the SRMSR (Hu and Bentler, 1999).

In Supplementary Materials (see Text Footnote 1; along with the Review of STEs), we included forest plots for a graphical depiction of the meta-analyses and the SEM diagrams with the effects of each emotion.

## Results

CFAs were conducted for the published structures of each scale and were performed in the congruent situations; that is, when the scale meets the prototypical stimulus for each particular emotion (Supplementary Table 2 for detailed information)^3^. With each CFA, adequate goodness-of-fit indexes were found for Awe (*X*^2^_(__73__)_ = 190.88, CFI = 0.962, RMSEA = 0.067, 95% CI [0.058, 0.067]), Elevation (*X*^2^_(__145__)_ = 395.85, CFI = 0.949, RMSEA = 0.071, 95% CI [0.064, 0.078]), and Kama Muta (*X*^2^_(__222__)_ = 551.48, CFI = 0.927, RMSEA = 0.064, 95% CI [0.058, 0.069]).

**FIGURE 2:**
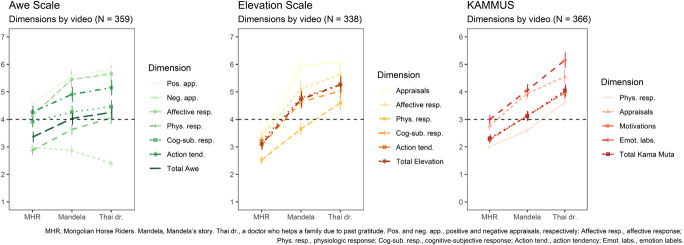
Emotional reactions to each video. Means and 95% CI for Awe **(Left)**, Elevation **(Center)**, and Kama Muta **(Right)** dimensions’ reactions to each video. The black dotted line from the y-axis represents the center point for each scale.

Observing the intensities of responses from the conditions regarding each video (Figure 2), we can observe that, as a whole, the Thai Dr. video was the one which induced the highest scores in every condition. While originally aimed at eliciting Kama Muta more strongly, this intensification of a communal sharing relationship also provoked the highest means for every condition –which is also a theme highly used in Gratitude and Elevation studies. In the case of the Awe condition (answering the Awe Scale), the responses with this scale increased with Mandela’s video and even more with the final ones. However, this condition had the highest scores of the nature-related video (above or closest to the scale’s midpoint). The tendency of the Elevation condition, while similar, had the lowest scores (as a whole) for the nature video and then, increased with Mandela and Thai Dr. videos. For Kama Muta, scores were gradually higher from the lowest (Mongolian Horse Riders) to the highest (Thai Dr.). Finally, for every condition, the self-reported physiological changes were generally the lowest, showing the highest responses (i.e., above the midpoint in each scale) only with the Thai Dr. video.

Correlation analyses (Tables 3–5) show that the total mean score of each emotion correlated significantly and positively with, Self-Transcendent Values, Helping Intention to Others, Identity Fusion with all humanity and Well-being. For each case, the strongest correlation was with Helping Intentions and the lowest, with Well-being. In detail, in the Awe condition, the strongest associations with the dependent variables were with the dimensions Cognitive-Subjective Response, and Action Tendencies, besides the Total of the scale (*r*s > 0.40). For Elevation, with Labels, Cognitive-Subjective Response, and Action Tendencies, besides the scale’s total (*r*s > 0.47). Finally, Kama Muta’s dimensions of Motivation, Labels, and Appraisals, besides the Total of the scale showed the strongest associations (*r*s > 0.30).

**TABLE 3:** Descriptive and correlation analyses of interest variables after answering the awe prototypical video.

Variable	*M*	*SD*	1	2	3	4	5	6	7	8	9	10	11	12
(1) Gender	–	–												
(2) Age	32.12	12.14	−0.12g**											
			[−0.18, −0.06]											
(3) Pos. app.	4.57	1.86	0.09	0.05										
			[−0.02, 0.19]	[−0.06, 0.15]										
(4) Neg. app.	2.98	1.85	−0.00	−0.06	0.09									
			[−0.11, 0.10]	[−0.17, 0.04]	[−0.01, 0.19]									
(5) Labels	4.14	1.78	0.06	0.05	0.83**	0.13*								
			[−0.04, 0.16]	[−0.06, 0.15]	[0.79, 0.86]	[0.03, 0.23]								
(6) Phy. Resp.	2.87	1.58	0.02	−0.05	0.52**	0.28**	0.64**							
			[−0.08, 0.12]	[−0.15, 0.06]	[0.44, 0.59]	[0.18, 0.37]	[0.58, 0.70]							
(7) Cog.-sub. resp.	3.92	1.70	0.09	0.01	0.68**	0.19**	0.80**	0.70**						
			[−0.02, 0.19]	[−0.10, 0.12]	[0.62, 0.73]	[0.09, 0.29]	[0.75, 0.83]	[0.64, 0.75]						
(8) Act. tend.	4.27	1.79	0.10	−0.03	0.65**	0.14*	0.74**	0.59**	0.85**					
			[−0.00, 0.20]	[−0.13, 0.08]	[0.58, 0.70]	[0.03, 0.24]	[0.69, 0.79]	[0.52, 0.66]	[0.82, 0.87]					
(9) Awe total	3.35	1.34	0.08	0.00	0.77**	0.20**	0.91**	0.80**	0.94**	0.89**				
			[−0.03, 0.18]	[−0.10, 0.11]	[0.72, 0.81]	[0.10, 0.30]	[0.89, 0.93]	[0.76, 0.84]	[0.93, 0.96]	[0.87, 0.91]				
(10) *S*-trans. values	5.15	0.76	0.22**	0.01	0.24**	0.01	0.27**	0.15**	0.29**	0.35**	0.30**			
			[0.16, 0.27]	[−0.05, 0.07]	[0.14, 0.33]	[−0.09, 0.11]	[0.17, 0.36]	[0.04, 0.25]	[0.19, 0.38]	[0.26, 0.44]	[0.20, 0.39]			
(11) Help intention	3.49	0.99	0.33**	−0.10	0.32**	0.11*	0.35**	0.26**	0.40**	0.40**	0.40**	0.36**		
			[0.23, 0.42]	[−0.20, 0.01]	[0.22, 0.41]	[0.00, 0.21]	[0.25, 0.43]	[0.16, 0.36]	[0.30, 0.48]	[0.30, 0.48]	[0.30, 0.48]	[0.27, 0.45]		
(12) Identity fusion	3.67	1.13	0.06	0.14**	0.25**	0.04	0.23**	0.19**	0.29**	0.27**	0.28**	0.19**	0.16**	
			[−0.05, 0.16]	[0.04, 0.24]	[0.15, 0.35]	[−0.07, 0.14]	[0.13, 0.33]	[0.09, 0.29]	[0.19, 0.38]	[0.17, 0.36]	[0.18, 0.37]	[0.09, 0.29]	[0.05, 0.26]	
(13) Well-being	8.31	1.86	0.03	−0.03	0.11*	0.07	0.14**	0.15**	0.22**	0.23**	0.21**	0.27**	0.16**	0.27**
			[−0.03, 0.09]	[−0.09, 0.03]	[0.01, 0.21]	[−0.03, 0.17]	[0.04, 0.24]	[0.05, 0.25]	[0.12, 0.32]	[0.13, 0.33]	[0.11, 0.31]	[0.21, 0.33]	[0.06, 0.26]	[0.17, 0.36]

M and SD are used to represent mean and standard deviation, respectively. For Gender, 1 = Male, 2 = Female. Pos. and neg. app., positive and negative appraisals, respectively; Phys. Resp., physiologic response; Cog-sub. resp., cognitive-subjective response; act. tend., action tendency; S-trans. Values, self-transcendent values. Values in square brackets indicate the 95% confidence interval for each correlation. * indicates p < 0.05. ** indicates p < 0.01.

**TABLE 4:** Descriptive and correlation analyses of interest variables after answering the elevation prototypical video.

Variable	*M*	*SD*	1	2	3	4	5	6	7	8	9	10	11
(1) Gender	–	–											
(2) Age	32.12	12.14	−0.12**										
			[−0.18, −0.06]										
(3) Appraisals	5.95	1.41	0.23**	0.09									
			[0.12, 0.33]	[−0.02, 0.19]									
(4) Labels	5.11	1.46	0.22**	0.06	0.71**								
			[0.11, 0.32]	[−0.05, 0.16]	[0.65, 0.76]								
(5) Phy. response	3.65	1.70	0.09	0.14*	0.39**	0.64**							
			[−0.02, 0.19]	[0.03, 0.24]	[0.29, 0.47]	[0.58, 0.70]							
(6) Cog.-sub. resp.	4.63	1.55	0.23**	0.05	0.54**	0.79**	0.65**						
			[0.12, 0.33]	[−0.06, 0.16]	[0.46, 0.61]	[0.74, 0.82]	[0.58, 0.71]						
(7) Act. tend.	4.78	1.61	0.18**	−0.00	0.53**	0.75**	0.55**	0.83**					
			[0.07, 0.28]	[−0.11, 0.11]	[0.45, 0.60]	[0.70, 0.79]	[0.47, 0.62]	[0.79, 0.86]					
(8) Elevation total	4.72	1.34	0.21**	0.08	0.69**	0.92**	0.79**	0.91**	0.88**				
			[0.11, 0.31]	[−0.03, 0.18]	[0.63, 0.74]	[0.90, 0.94]	[0.75, 0.83]	[0.89, 0.93]	[0.85, 0.90]				
(9) *S*-trans. values	5.15	0.76	0.22**	0.01	0.34**	0.41**	0.26**	0.40**	0.36**	0.41**			
			[0.16, 0.27]	[−0.05, 0.07]	[0.24, 0.43]	[0.31, 0.49]	[0.16, 0.36]	[0.31, 0.49]	[0.27, 0.45]	[0.32, 0.50]			
(10) Help intention	3.44	0.94	0.33**	−0.03	0.34**	0.47**	0.33**	0.50**	0.48**	0.50**	0.39**		
			[0.23, 0.42]	[−0.14, 0.08]	[0.24, 0.43]	[0.38, 0.55]	[0.23, 0.42]	[0.41, 0.57]	[0.39, 0.56]	[0.42, 0.58]	[0.29, 0.47]		
(11) Identity fusion	3.66	1.09	0.08	0.15**	0.19**	0.24**	0.21**	0.29**	0.27**	0.29**	0.17**	0.21**	
			[−0.03, 0.19]	[0.04, 0.26]	[0.08, 0.29]	[0.14, 0.34]	[0.11, 0.31]	[0.19, 0.38]	[0.17, 0.36]	[0.18, 0.38]	[0.06, 0.27]	[0.11, 0.31]	
(12) Well-being	8.31	1.86	0.03	−0.03	0.11*	0.14**	0.03	0.13*	0.14**	0.13*	0.27**	0.10	0.28**
			[−0.03, 0.09]	[−0.09, 0.03]	[0.01, 0.22]	[0.04, 0.25]	[−0.07, 0.14]	[0.02, 0.23]	[0.04, 0.25]	[0.02, 0.23]	[0.21, 0.33]	[−0.01, 0.20]	[0.18, 0.37]

M and SD are used to represent mean and standard deviation, respectively. For Gender, 1 = Male, 2 = Female. Phys. response, physiologic response; Cog-sub. resp., cognitive-subjective response; Act. tend., action tendency; S-trans. Values, self-transcendent values. Values in square brackets indicate the 95% confidence interval for each correlation. * indicates p < 0.05. ** indicates p < 0.01.

**TABLE 5:** Descriptive and correlation analyses of interest variables after answering the Kama Muta prototypical video.

Variable	*M*	*SD*	1	2	3	4	5	6	7	8	9	10
(1) Gender	–	–										
(2) Age	32.12	12.14	−0.12**									
			[−0.18, −0.06]									
(3) Phy. response	3.63	1.38	0.10*	0.06								
			[0.00, 0.20]	[−0.04, 0.17]								
(4) Appraisals	4.55	1.68	0.06	0.06	0.68**							
			[−0.04, 0.16]	[−0.05, 0.16]	[0.62, 0.73]							
(5) Motivations	3.97	1.79	0.09	−0.04	0.61**	0.74**						
			[−0.01, 0.19]	[−0.14, 0.06]	[0.54, 0.67]	[0.69, 0.78]						
(6) Labels	5.15	1.43	0.09	0.05	0.77**	0.75**	0.61**					
			[−0.02, 0.19]	[−0.05, 0.16]	[0.73, 0.81]	[0.70, 0.79]	[0.54, 0.67]					
(7) KM total	4.04	1.34	0.10*	0.04	0.94**	0.86**	0.81**	0.86**				
			[0.00, 0.20]	[−0.06, 0.15]	[0.92, 0.95]	[0.83, 0.89]	[0.77, 0.84]	[0.83, 0.88]				
(8) *S*-trans. values	5.15	0.76	0.22**	0.01	0.25**	0.37**	0.24**	0.31**	0.31**			
			[0.16, 0.27]	[−0.05, 0.07]	[0.15, 0.34]	[0.27, 0.45]	[0.14, 0.33]	[0.21, 0.40]	[0.21, 0.40]			
(9) Help intention	3.41	1.00	0.22**	−0.09	0.31**	0.37**	0.30**	0.33**	0.37**	0.39**		
			[0.12, 0.32]	[−0.19, 0.02]	[0.21, 0.40]	[0.28, 0.46]	[0.21, 0.39]	[0.24, 0.42]	[0.27, 0.45]	[0.30, 0.48]		
(10) Identity fusion	3.65	1.09	0.01	0.12*	0.26**	0.36**	0.24**	0.27**	0.31**	0.17**	0.26**	
			[−0.09, 0.11]	[0.01, 0.22]	[0.17, 0.36]	[0.26, 0.44]	[0.15, 0.34]	[0.17, 0.36]	[0.22, 0.40]	[0.07, 0.27]	[0.16, 0.35]	
(11) Well-being	8.31	1.86	0.03	−0.03	0.13*	0.18**	0.08	0.11*	0.14**	0.27**	0.10	0.17**
			[−0.03, 0.09]	[−0.09, 0.03]	[0.03, 0.23]	[0.08, 0.28]	[−0.02, 0.18]	[0.01, 0.21]	[0.04, 0.24]	[0.21, 0.33]	[−0.00, 0.20]	[0.07, 0.27]

M and SD are used to represent mean and standard deviation, respectively. For Gender, 1 = Male, 2 = Female. Phy. response, physiological response; KM total, Kama Muta total; S-trans. Values, self-transcendent values. Values in square brackets indicate the 95% confidence interval for each correlation. * indicates p < 0.05. ** indicates p < 0.01.

Subsequently, the random effects model used to meta-analyze the correlation between the manifestation of each emotion and the dependent variables (Table 6) showed that each emotion does relate significantly with Identity Fusion with the Humanity and Helping Intention to Others, being the pooled effects stronger with the latter. In addition, heterogeneity tests indicate these relationships are invariant across the contexts. In the relationship with Well-being, on the contrary, pooled *r*s were non-significant and analyses revealed significant sources of heterogeneity. In other words, that the relationships are affected by others factors. In all, these analyses suggest that these emotions may not necessarily be directly associated with participants Well-being and in general, most of the relationships among the variables are invariant across the regional contexts.

**TABLE 6:** Random-effect meta-analyses of the effects of each emotion on ID fusion with humanity, help intention to others, and individuals wellbeing.

		Video and scale					
		Awe (*n* = 359) *Mongolian Horse Riders*		Elevation (*n* = 338) *Mandela’s Life*		Kama Muta (*n* = 366) *Thai Dr.*	
Dependent	Predictor	*r*_*pooled*_ [95%CI]	Heterogeneity *Q*_(2)_, *I*^2^	*r*_*pooled*_ [95%CI]	Heterogeneity *Q*_(2)_, *I*^2^	*r*_*pooled*_ [95%CI]	Heterogeneity *Q*_(2)_, *I*^2^
ID Fusion	Emotion^1^	**0.294** [0.209, 0.379]	3.898, 46.20%	**0.280** [0.224, 0.335]	0.586, 0.0%	**0.288** [0.200, 0.377]	0.586, 0.0%
Help intention	Emotion^1^	**0.391** [0.331, 0.452]	2.008, 17.49%	**0.516** [0.471, 0.560]	1.388, 0.0%	**0.382** [0.331, 0.433]	2.468, 0.0%
Wellbeing	Emotion^1^	0.190 [−0.049, 0.429]	**23.067**, 92.90%	0.145 [−0.000, 0.291]	**10.424**, 78.61%	0.082 [−0.115, 0.279]	**11.315**, 88.02%

*^1^Each emotion, for every model, is the target emotion that is matched with the prototypical stimulus. Bold estimates indicate significant values at p < 0.05. ID Fusion, Identity Fusion. Forest plots of these analyses can be seen in Supplementary Materials online.*

Finally, we examined separated SEM models as they are shown in Figure 1. In each model (Table 7), we found overall adequate fit indexes and similar patterns among some relationships. First, we can see that, when predicting Identity of Fusion with all humanity, each STE positively and significantly predicts it, while Self- Transcendent Values do not. This emotional effect is maintained when the dependent variable is Helping Intentions to others, along with positive and significant coefficients for Values – and ID fusion, in the case of Kama Muta. Finally, when predicting participants Well-being, we see that only Awe significantly explains it (for Kama Muta, *p* = 0.075). In addition, subsequent exploration of indirect effects revealed that, each STE increases participants Well-being indirectly through higher scores in Identity Fusion with all humanity (*B* = 0.72, *p* = 0.007; *B* = 0.127, *p* = 0.004; and *B* = 0.062, *p* = 0.024).

**TABLE 7:** SEM analyses predicting ID fusion with humanity, help intention to others, and individuals wellbeing.

		Video and scale								
		Awe (*n* = 359) *Mongolian Horse Riders*			Elevation (*n* = 338) *Mandela’s Life*			Kama Muta (*n* = 366) *Thai Dr.*		
Dependent	Predictor	*B*	*SE*	95% CI	*B*	*SE*	95% CI	*B*	*SE*	95% CI
ID fusion	Emotion^1^	**0.270**	0.058	[0.157, 0.383]	**0.275**	0.068	[0.142, 0.409]	**0.303**	0.052	[0.202, 0.405]
	ST values	0.113	0.075	[−0.033, 0.259]	0.058	0.071	[−0.081, 0.198]	0.077	0.053	[−0.027, 0.182]
Help intention	Emotion^1^	**0.367**	0.055	[0.259, 0.475]	**0.429**	0.069	[0.293, 0.565]	**0.238**	0.064	[0.113, 0.362]
	ST values	**0.276**	0.066	[0.147, 0.406]	**0.252**	0.065	[0.125, 0.379]	**0.326**	0.063	[0.202, 0.451]
	ID fusion	−0.035	0.060	[−0.152, 0.082]	0.035	0.053	[−0.069, 0.139]	**0.135**	0.054	[0.029, 0.240]
Wellbeing	Emotion^1^	**0.246**	0.064	[0.121, 0.372]	0.151	0.089	[−0.022, 0.325]	0.145^†^	0.073	[0.002, 0.288]
	Help. intention	0.069	0.085	[−0.097, 0.236]	0.032	0.087	[−0.139, 0.203]	0.086	0.075	[−0.061, 0.232]
Models’ fit	*X*^2^ (*df*)	627.77 (288)			903.92 (422)			1123.48 (545)		
	CFI	0.935			0.920			0.918		
	TLI	0.927			0.911			0.911		
	RMSEA [IC90%]	0.057 [0.052, 0.063]			0.058 [0.053, 0.063]			0.054 [0.050, 0.058]		
	SRMR	0.064			0.058			0.062		

*B and SE represent standardized SEM regressions coefficients and standard errors, respectively. Bold estimates indicate significant values (p < 0.05; ^†^, p < 0.10). ^1^Each emotion, for every model, is the target emotion that is matched with the prototypical stimulus. ID fusion, identity fusion; ST values, self-transcendent values. Models’ fin indexes are Chi-square (X^2^), df, degrees of freedom; CFI, comparative fit index; TLI, Tucker–Lewis index; RMSEA, root mean square error of approximation; SRMR, standardized root mean square residual.*

## Discussion

Answering unequivocally to the questions presented at the beginning of this manuscript is not a simple task. However, this research shows that a possible way is through STEs. In other words, that experiencing STEs increase people’s sense of identification with the humanity, the willingness of collectively help others and participants’ well-being. The path observed is stable through different stimuli and multi-component scales, and all converge in suggesting a common profile of ST (Van Cappellen and Rimé, 2014; Stellar et al., 2017b). This profile (i.e., orientation to the needs of others, even strangers) becomes even more important if we consider the context in which these emotions are produced, and the situational nature of them. Specifically, the relevance of the context lies in the fact that the effects presented here take place from individual experiences that have subsequent implications for collective behaviors. Regarding the nature of these emotions, on the other hand, because these effects are being proved to result from situational experiences (i.e., STEs), and not uniquely from individual orientations to help and/or identify with other people (i.e., ST values).

Regarding H1, the results show that the emotional reactions in the congruent conditions (i.e., prototypical stimulus and scale) were not as predicted for the case of the Awe scale, and in part, for the Elevation scale. In detail, the Awe scale (Pizarro et al., 2018) reacted more strongly to the moral figure of Mandela (i.e., prototypical for Elevation) and even more strongly to an intensification of a communal sharing relationship (i.e., Kama Muta prototypical stimulus). This suggests that social stimuli that emphasize benevolence, morality and intense relationships based on solidarity and unity (i.e., communal sharing) are stronger –compared to nature– elicitors of an emotional reaction characterized by a sense of grandness and amazement (Keltner and Haidt, 2003; Pizarro et al., 2018). In the case of Elevation, on the other hand, the pattern observed is more congruent with the Elevation framework (e.g., Haidt, 2000). In specific, the Elevation scale (Cusi et al., 2018) performed somewhat similarly and the strongest reactions were found in the communal sharing video, which emphasizes solidarity and helping as form of solidarity in connection. Finally, for Kama Muta, the KAMMUS-Two scale (Zickfeld et al., 2018) showed an activation pattern in full concordance with the Kama Muta framework (see Fiske et al., 2017b). In all, even though we were not able to fully distinguish these three emotions (see the following section), the reactions measured to the prototypical stimuli did show effects in line with the theorization of STEs.

These effects can be seen in the congruent conditions from where the main analyses were conducted. In detail, the focus on magnificent landscapes and nature, the history of Mandela, and an intensification of communal sharing relationships were proved to provoke a ST pattern in the form of willingness to engage on collective action and to identify to a superordinate identification. Besides supporting the ST hypothesis, these results have important implications for the study of collective action and the study of global identity, because it present a path that has not been largely developed.

When considering extended frameworks of collective action such as the SIMCA model (van Zomeren et al., 2019) and the EMSICA (Thomas et al., 2009), one can observe that these models explicitly treat emotional experiences as pre-requisites for a shared identification and its further enhancement of collective action. In addition, they usually focus on negative affect, such as moral outrage (e.g., Thomas et al., 2009). In contrast, the present results from STEs propose a different yet complementary approach. Here, the results show that STEs can directly motivate both intentions to engage on collective action and at the same time, a common and shared identity that in all, are more congruent with a theorization of ST experiences (Stellar et al., 2017b; Yaden et al., 2017). This form, the social functionality of these emotions is seen in motivating individuals toward others’ needs and provoking a sense of connection to them independently, rather than sequentially. What is more, the themes used in eliciting them (i.e., nature, a moral leader, and intense connection and solidarity) are highly used in different social movements all over the world. Therefore, even though our study was conducted “in the lab,” we could also hypothesize about their presence in ongoing forms of collective practices, since they are also proved as elicitors of these emotions in the form of social gatherings in the form of recalling eliciting events for Awe (Pizarro et al., 2018; Pizarro, 2019). Furthermore, these emotions can also center the attention on the role of positive affect in analyzing the motivators in the study of collective action, where the general attention has been usually centered on negative emotions (e.g., anger).

Finally, we found that the effects of STEs on participants Well-being are more complex in nature. While there are indeed previous studies showing these links (e.g., Van Cappellen et al., 2014), we find (a) significant sources of heterogeneity, while (b) direct significant correlations and indirect SEM effects through a fusion of identity with all humanity. In all, we consider that this could be in part of two main reasons. The first, due to the limitation regarding measurement; that is, measuring a more stable facet of Well-being, compared to other measures (see Curry et al.’s meta-analysis). We consider this clearly affected the results because affective states are more proximal than psychological or cognitive Well-being, such as satisfaction with life. On the other hand, the discrepancies could be –at least– partially explained by the contexts participants were from. However, it is worth mentioning that all the three STEs affected Well-being indirectly through more intense fusion of identity with all humanity.

In all, the results show how these emotions can explain intentions to collectively help others (i.e., Elevation, Awe, and Kama Muta, in descending order), to psychologically feel connected with humanity (i.e., Kama Muta, Awe, Elevation, in descending order), and also, they produce an increase in people’s well-being.

### Alternative Hypotheses

Considering competing hypotheses for the results found, one could be that the three STEs used here are intrinsically similar manifestations of an underlying emotion. Therefore, they react in a highly similar way across different stimuli. Nevertheless, at least in the case of Kama Muta and Awe, Zickfeld et al. (2018) have shown particular differences in the responses of both emotions across a large sample (*N* = 3,542) in 15 different languages. Further, the reactions in the intensity of responses to the nature-related video shows that the Awe scale more intensively react to it, compared to the responses of Elevation and Kama Muta. Even though that video did not produce the strongest intensities in Awe, it provoked a similar pattern congruent to ST theorizations.

Another alternative hypothesis was centered on the idea that only the human identification was the motivator of collective action intentions. In other words, that the three STEs motivate a superordinate identification, and through that, the helping intention in the form of collective action is possible. Even though the path including a superordinate identity and prosociality has been demonstrated (e.g., Buchan et al., 2017; McFarland et al., 2019), the present study clearly shows direct effects of these emotions on collective action intentions. This suggests that, in the case of the activation of a superordinate identity, helping intentions are not intrinsically identity-dependent. Rather, this identification might work as another path increasing the intention to help and/or a direct effect of these emotions.

### Limitations

The present study was not exempt of limitations. Of most relevance, there was not any control condition and therefore, causality can only be suggested. Even though these effects are produced with a video elicitation, the present study is correlational in nature and there is still the need to include several control groups (i.e., ideally, with other positive emotions and without any emotional reaction). In addition to this, the measure used to evaluate well-being was not optimal because it was oriented at measuring a more stable facet of well-being (i.e., satisfaction with life), and we conducted the study with university students, which correspond to samples that cannot be directly generalized to the whole population (see Henrich, 2020). Finally, it would have been appropriate to analyze other types of individual differences, different from the ST values of the Schwartz model.

There is no doubt that these results would be more robust if these limitations had been minimized or eliminated. Even so, the findings of this proposal are in full agreement with the theoretical proposal and therefore, we consider them to be of great value for the study of human emotions.

### Future Perspectives

Focusing on different forms of positive affect under the form of STEs can be a promising research line, particularly in the framework of collective action and the study of human identities. Besides the highlighted limitations, the following lines present fruitful guidelines for future works. To begin, the incorporation of individual differences and other factors that might affect ST experiences. This is because the proved effects of STEs could work as amplifiers of personal dispositions. To illustrate, the themes eliciting STEs (i.e., nature, morality, and strong solidarity-based links) are themes present in different forms of social protests and demonstrations. This way, when measuring emotional reactions in these contexts, these particular emotions could magnify individual tendencies (e.g., trait of openness to experience, or measures of interpersonal empathy as it has been shown before; see Zickfeld et al., 2018). Consequently, if we incorporate different measures of individual dispositions, we can better elucidate the role of emotions (i.e., situational factors) and traits (i.e., individual differences).

In addition to individual differences, there is role of self-compassion, a psychological construct that can affect the form we experience a STE. Although it is different from feeling compassion to others, this construct entails implications for one’s well-being as well as the recognition of a common humanity (Neff, 2011). In addition, it can affect how we process prototypical ST stimuli, such as those connected to other’s suffering, inter-personal help, and compassionate responses (see Yaden et al., 2017).

Finally, we consider that future works should provide multi-component approaches the study of emotions as well as the incorporation of naturally occurring events. Multi-component approaches ensure that STEs measurements are adequate. Due to a high anchoring in language use, several studies have attempted to differentiate STEs (e.g., Onu et al., 2016) with a high dependence on the use of language. Unfortunately, emotional words that do not necessarily correspond neither to a clear emotion, nor to an experience that is replicated in other contexts. In relation to the study of naturally occurring events, on the other hand, it can help to maximize the validity of theoretical models of these emotions; especially since there is a wide range of contexts, in which STEs can arise.

While some have been employed in the study of STEs (e.g., nature use, see Bai et al., 2017; on communication and social media, see Oliver et al., 2015), there are several understudied such as those where interpersonal relationships are essential. A clear example is educational settings. In these contexts, STEs such as wonder and moral inspiration can naturally arise in student-teacher relationships and are likely to affect the outcomes of the learning process. In addition, the people who may provoke these emotions in their students are undoubtedly affected by other variables described above, such as self-pity (see Moè and Katz, 2020), which makes this context of greater interest.

In all, we believe that this line of study can produce research with applicable results to a wide range of contexts.

## Data Availability Statement

The datasets generated for this study can be found in online repositories. The names of the repository/repositories and accession number(s) can be found in the article/Supplementary Material.

## Ethics Statement

The studies involving human participants were reviewed and approved by UPV/EHU CEISH, No Ref CEID: M10/2016/031. The patients/participants provided their written informed consent to participate in this study.

## Author Contributions

JP, NB, and DP conceptualized the first project, which was further developed by IF and PC. The investigation was then carried by JP, PA, and CM. All statistical analyses and (as well as visualizations) were carried out by JP with NB, OC, PA, and DP. JP wrote the first draft of the manuscript, which was then reviewed and accepted by all authors.

## Conflict of Interest

The authors declare that the research was conducted in the absence of any commercial or financial relationships that could be construed as a potential conflict of interest.

## Publisher’s Note

All claims expressed in this article are solely those of the authors and do not necessarily represent those of their affiliated organizations, or those of the publisher, the editors and the reviewers. Any product that may be evaluated in this article, or claim that may be made by its manufacturer, is not guaranteed or endorsed by the publisher.

## Footnotes

1
https://osf.io/vbrme/

2 The sample size was calculated *a priori* using the software G^∗^Power (Faul et al., 2007) and using two different criteria. The first, predicting a multiple linear regression predicting the dependent variables for each condition: two-tailed, with an effect size (*f*^2^) = 0.15, α = 0.01, power = 0.90, and accounting for 4 predictors = 4, the analysis showed a resulting sampling of 103 participants (for condition) and 309 in total. The second analysis, predicting goodness-of-fit for each condition, was based on a predicted effect size (*W*) = 0.40, α = 0.05, power = 0.90, and degrees of freedom = 100, which results in 309 participants (for condition) and 927 total. Taken both criteria into account, we stopped data collection when we surpassed the 1000 participant limit. Additionally, we conducted *a posteriori* power analysis based on SEM analysis and following the guidelines of Gnambs (2019, based on Kim, 2005), which showed that the minimum total sample should have been 588 (with α = 0.05, power = 0.80).

3 Conducting analyses with the congruent scenarios was considering the most appropriate way to test the hypothesis of the present study. Not only for the CFAs, but also for hypotheses H2–H4, were we can guarantee that the effects –if any– are present across different stimuli and scales. In addition, we invite researchers to explore the dataset and syntax freely available at https://osf.io/vbrme/.
