# Supplementary material for: Self-Transcendent Emotions and Their Social Effects: Awe, Elevation and Kama Muta Promote a Human Identification and Motivations to Help Others
Source: Front Psychol. 2021 Sep 13;12:709859. doi: 10.3389/fpsyg.2021.709859 (PMC8473748; doi:10.3389/fpsyg.2021.709859)
Supplement: Supplementary file 1 [file Data_Sheet_1.pdf]

Self-Transcendent Emotions and their Social Effects: Awe, Elevation and Kama Muta Promote a Human Identification and Motivations to Help Others

Review of Effects of STEs

Supplemental Table S1 presents a non-systematic review of studies that analyse the effects of 5 different STEs on direct or indirect forms of connecting to other people. The emotions selected were Awe, Elevation, Gratitude, Compassion, and Kama muta. It indicates the type of stimuli used in each study, the effect, and finally, the type of effects that implies (i.e., direct, indirect, or both) for connecting with others.

This review included studies up to 2020 that empirically produce or measure each STE and at least one form reflecting an inter-personal or inter-group connection. The review was conducted examining psychology-related databases (i.e., PsycINFO, SCOPUS, WoS) and finally, Google Scholar. The search criteria used were the name of these STEs and the languages included were English and Spanish.

Supplemental Table S1.

Review of the Effects of STEs on the Connection with Others.

Emotion	Reference	Study, elicitors or measurement of interest	Effects on connection to others	Effect on behaviour	
				Direct	Indirect
Awe	Shiota et al. (2007).	S2: recalling event.	Greater perceptions of feeling small and connected to something bigger than one (e.g., the world around) feels.		X
	Schurtz et al. (2012).	S3: recalling event.	More frequency of self-reported goose bumps and a greater sense of vastness.		X
	Van Cappellen and Saroglou (2012).	S1: recalling event.<br>S2: video of nature.	Greater willingness to visit a spiritual destination (S1), and to connect to others (S2), through the increase of religiosity and spirituality.		X
	Piff, Dietze, Feinberg, Stancato, and Keltner (2015).	S2: recalling event.<br>S3: video of nature (from BBC's *Planet Earth*).<br>S4: video of threatening phenomena, and coloured droplets in slow motion (from *The Slow Mo Guys*).	An increase of ethical thinking and helping behaviour (S2), more generosity in a dictator game (S3), and increased prosociality for resource allocation (in both awe conditions in S4).	X	X
	van Elk, Karinen, Stamkou, and Baas (2016).	S3: video of nature (from BBC's *Nature*).	Decreased perception of body size (i.e., more pronounced for those with higher scores in absorption in the video) (S3).		X

Joye and Dewitte (2016).	PS1b: computer-created building pictures.<br>S1b: building pictures.	Greater behavioural freezing (PS1b and S1b).		X
Yang, Yang, Bao, Liu, and Passmore (2016).	S1, S2: recalling event.<br>S3: video of nature (from BBC's *Planet Earth*).	Decreased self-reported aggressive behaviour (S1, S2, S3), and greater prosocial behaviour and motivations (S3).	X	X
Bai et al. (2017).	S2: daily reports.<br>S3: Yosemite national park (field study).<br>S4, S5: video of nature (from BBC's *Planet Earth*).<br>S6: recalling event.	Greater perception of smaller self-size during the days when awe is more strongly felt (S2), when in a natural park (S3), and in the lab (S4, S5, S6). Also, a greater sense of connection with others (S6).		X
Stellar, Gordon, Anderson, et al. (2017).	S2: daily reports.<br>S3: video of space.<br>S4: recalling event.<br>S5: monuments and city views (field study).	More reported humility the days of experiencing awe (S2), less self-enhancement (i.e., less personal strengths in S3, and more external forces in S4), and more humility (S5).		X
Pizarro et al. (2018).	S2: list of events and recalling event.	Greater associations with universality beliefs and identification with a global identity.		X
Guan, Chen, Chen, Liu, and Zha (2019).	S2, S3: video of nature, to elicit positive (S2, S3) and negative awe (only S3) (both from BBC's *Planet Earth*).	More intention to donate to help person in need (S2) and to help another researcher with the participants' time (S3).	X	
Nelson-coffey, Ruberton, Chancellor, and Cornick (2019).	S2, S3: video of the earth zooming away with a selection of reads from Carl Sagan's book *Pale Blue Dot*.	Increased feelings of connectedness with all humanity and of feeling small	X	
Johnson et al. (2019).	S2: video of six famous physicists marvelled by quantum and particle physics (*Symphony of Science – the Quantum World!*)<br>S3: a video centred on the immensity of the universe (*The Wonder of the Universe*) and the complexity of a cell (*The Wonder of a Living Cell*)	Greater agreement with transcendent beliefs, which in turn, and different forms of mentally representing God, such as *mystical* (i.e., described as *nature* or *cosmic*), or *ineffable* (i.e., described as *unknowable* or *incomprehensible*).		X

Elevation	Silvers and Haidt (2008).	Video of a person paying tribute to a teacher (from the *Oprah Winfrey Show*).	Greater caring behaviour (mothers with their children).	X	
	Algoe and Haidt (2009).	S2a: boy who established a homeless shelter.<br>S2b: daily reports.	Tendency to emulate moral actions, to be prosocial (S2a and S2b).		X
	Freeman, Aquino, and McFerran (2009).	S1: video of intergroup help (from *60 Minutes II*).<br>S2: reading news of forgiveness.	More disposition to donate a Black-oriented charity, and a decrease in group-based dominance (for those who had it high…over-ride it), and actual donating behaviour.	X	X
	Schnall et al. (2010).	S1 and S2: video of a person paying tribute to a teacher (from the *Oprah Winfrey Show*).	Greater helping intention and improve personally (S1), and helping behaviour (S2).	X	X
	Cox (2010).	Spring-break service trip (naturalistic study).	Greater frequency of self-reported times of having participated in similar voluntary work (1 week and 3 months after).	X	
	Vianello, Galliani, and Haidt (2010).	S1: reading fictional leaders' descriptions.<br>S3: recalling event.	Increased Organizational Citizenship Behaviour (OCB), more affective commitment to the organization (S1 and S2), and more willingness to behave altruistically (S2).		X
	Aquino, Mcferran, and Laven (2011).	S2: recalling event.<br>S3: priming of moral identity and reading news of forgiveness.<br>S4: video of donation to several charities (*World on Fire*).	S2: elevation emotions were associated to a greater motivation to help others<br>S3: greater prosocial behaviour (modified dictator game).	X	X
	Oliver, Hartman, and Woolley (2012).	Naming and rating a meaningful film they had watched (recalling).	Greater agreement with altruistic values, increased feelings of wanting to become a better person and do good things for others.		X
	Thomson and Siegel (2013).	S2: reading a story of helping a person that was *moral* or *immoral*.<br>S3: recalling event of witnessing helping behaviours to different people.<br>S4: imaging a story of one person helping other with different effort.	Increased donations (to a children charity) from the money participants received (S2). In addition, this help was greater when the behaviour was toward a person who needed (S3), and to when it was more difficult (S4).	X	X

Thomnson, Nakamura, Siegel, and Csikszentmihalyi (2014).	Reading a story of moral excellence.	More positive attitudes and mentoring behaviours.	X	X
Lai, Haidt, and Nosek (2014).	S1-4: video of a person paying tribute to a teacher (from the *Oprah Winfrey Show*).<br>S2-4: video of example of sportsmanship.<br>S3-4: news video of a person who saved another.	Reduction of implicit and explicit prejudice towards a sexual minority (i.e., gay men) (S1-4).		X
Siegel, Thomson, and Navarro (2014).	S1-S3: recalling event.	More positive views on humanity (S1), and greater donations to a children charity (S2 and S3).	X	X
Oliver et al. (2015).	Watching one of two inspiring videos (a person offering free hugs, or street musicians performing simultaneously).	Increased perceptions of shared goodness, greater overlap self-humanity, and more positive attitudes towards diverse groups of immigrants.		X
Van de Vyver and Abrams (2015).	S1 and S2: video of forgiveness to a perpetrator.	More donations to charity (S1), and increased prosocial motivations to engage in political actions (S2).	X	X
Erickson et al. (2017).	S1-S3: videos of virtuous actions (daily sent).	Less self-image related goals (S1-S3), and more compassionate-related goals (S1 and S2).		X
Tingey, Mcguire, Stebbins, and Erickson (2017).	Recalling helping behaviours subsequent to a school shooting in the US.	Elevation predicted increased perceptions of having been more oriented to others (i.e., helping, comforting) and post-traumatic growth after the shooting (the latter, after 8 months).	X	X
Cusi et al. (2018).	S2: list of events and recalling event.	Greater associations with universality beliefs and identification with a global identity.		X
Ding et al. (2018).	Recalling event.	More intentions to help, manifested in the time participants would dedicate to help another researcher –also, higher among those with a stronger moral identity.	X	

	Yao and Enright (2018).	S1, S2: reading different stories of people helping others.	Increased prosocial intentions (S1), and the intention to donate what they might win after completing the study (S2).	X	
	Rieger, Frischlich, and Oliver (2018).	Recalling event of having watched a meaningful movie.	Elevation reported for the recalled event predicted feelings of self-transcendence and subsequently, increased moral motivations.	X	
	Ellithorpe, Huang, and Oliver (2019).	S1, S2: watching an elevating video (political speech).	Higher intentions of participating in politics and to know more about who gave it (S1); it is replicated in study 2 (regardless the political orientation of the person who gave it), as well as a greater feelings of closeness of both ingroup and outgroup members.	X	
	Pohling, Diessner, Stacy, Woodward, and Strobel (2019).	Watching a morally uplifting video of a humble person helping others.	Higher amount of money allocated in the dictator's game (compared to control).	X	
	Zhang, Chen, Tao, Farid, and Ma (2019).	Participating in a public goods dilemma with a confederate who always contributed to the environment.	Increased amounts in the tokens participants would give to the environment fund, compared to control.	X	
Gratitude	Emmons and McCullough (2003).	S2: 16-day of daily reports of gratitude.	Greater self-reported likely of having provided emotional support (S2).	X	
	Barlett and DeSteno (2006).	S1, 2: Receiving help from a confederate so as to avoid a tedious task.	Measured in minutes of helping with a tedious task the person from which help was received (S1), and from strangers (S2).	X	
	Tsang (2006).	Having received a money during a money-distribution task.	More money allocated to their partners and a greater motivation to express appreciation .	X	X
	Algoe, Haidt, and Gable (2008).	4-day program where mentors give presents to new members of a sorority.	Benefactor responsiveness predicted gratitude which predictive future outcomes in the relationship (feelings of integration).	X	
	Algoe and Haidt (2009).	S3: write a letter to tell when someone did good to them (gratitude).	Intention to give back something; greater likelihood of interacting with the moral model.		X

Lambert, Clarck, Durtschi, Finchman, Graham (2010).	S3: Individual expressions of gratitude to a partner (diary study).	Increased communal strength (i.e., motivations to attend the needs of a partner).		X
DeSteno, Barlett, Baumann, Williams, and Dickens (2010).	Receiving help from a confederate so as to avoid a tedious task.	More money given to benefactors and strangers (money allocation task).	X	
Algoe, Gable, and Maisel (2010).	Daily accounts of gratitude to and from participants' romantic partners.	Increased satisfaction in relationship and subjective connection with one's partner.		X
Lambert and Fincham (2011).	S4: 3-week Program to increase the frequency to express gratitude to a friend.	A more positive perception of a friend, and more comfortable for addressing concerns.		X
Algoe and Stanton (2012).	Recalling person and event of gratitude.	Increased perceived social support (i.e., among women with low ambivalence over emotional expression).		X
Lambert, Fincham, and Stillman (2012).	S5: Think and write about ones opportunities and blessings<br>S7: gratitude journal.	Less self-reported depressive symptoms (study 5); S7: (longitudinal) more positive emotions.		X
Algoe, Fredrickson, and Gable (2013).	Expressing gratitude to one's partner.	Association of perceived responsiveness of an expression of gratitude with happiness (T1 and T2, after 6 months).		X
Williams and Barlett (2014).	Receiving a note expressing gratitude in a mentoring program.	Perceived writers as more appreciative, warmer, higher affiliative intentions, and more people leaving contact information.	X	X
O'Leary and Dockray (2015).	Having a gratitude diary (listing and guided gratitude reflection).	Pre-post: less stress, depression, and more happiness (intraindividual variables).		X
Algoe, Kurtz, and Hilaire (2016).	S1, S2: expressing gratitude to their partners.	More self-reported positive emotions (among them, love) and expresser's responsiveness (S1 and S2).		X
Tsang and Martin (2017).	S3: Having received a present in a resource-distribution task.	More resources allocated to their partners and a greater expression of gratitude.		X

	Bock, Eastman, and Eastman (2018).	Cross-sectional survey with participants who had volunteered or donated within the previous year.	Greater values motivation (i.e., concern of others) and helping intention to donate to charity.		X
	Vayness and DeSteno (2018).	Recalling event of gratitude.	Greater third party punishment (in a dictator's game).		X
Compassion	Sprecher and Fehr (2005).	S1-S3: self-reported measures of compassion (correlational).	Association with empathy (S1), intention to help others (S2), and social support offered to close people (S3).	X	X
	Sprecher, Fehr, and Zimmerman (2007).	Imagining having given or received a gift from others.	Compassion associated to expectations of feeling positive emotions, and interpersonal caring behaviors (e.g., verbal support, expression of empathy) (in both conditions).	X	X
	Crocker and Canevello (2008).	Weekly accounts of compassionate goals (diary study).	Compassionate goals were ssociated to beliefs of interconnectedness of people, less zero-sum views of succes, and more social support, trust and beliefs in mutual caring, regarding other students.		X
	Condon and DeSteno (2011).	Observing a person crying due to her brother's medical condition.	Lesser punishment to a person who cheated and showed no remorse.	X	
	Valdesolo and DeSteno (2011).	Manipulating behavioral synchrony with a confederate.	Increased compassion for the a victim of a fairness-related transgression, which in turn, led to helping them for longer periods of time.	X	
	Sinclair, Fehr, Wang, and Regehr (2016).	S1 and S2: self-reported measures of compassion (correlational).<br>S3: recalling event (selflessly gave of themselves to help others).<br>S4: self-reported measure of compassion (correlational).	Negative associations between compassion and prejudice towards different social groups (S1 and S2), less prejudice (S3), and less intention to deduct money from an immigrant fund (S4).	X	
	Lim and DeSteno (2016).	S1: self-reported measure of dispositional compassion (correlational).<br>S2: observing an ill person completing a tedious task.	Greater intentions of donations to a charity (S1), and more time helping a person with a tedious task (S2).	X	X

Kama Muta	Zickfeld and Schubert (2018).	Pictures of tearful people.	Increased kama muta (measured as feeling moved), which mediated how warm people were evaluated.		X
	Zickfeld et al. (2018).	Videos of intensifications of Communal Sharing relationships (Fiske, 1991).	Greater associations with empathic concern to others (as a trait).		X
	Seibt, Schubert, Zickfeld, and Fiske (2018).	S1 and S2: Videos of emotionally moving political campaigns.	Greater intention to support the political candidate (S1 and S2).		X
	Pierre (2019).	Analyses of a sample of Facebook posts on social movements.	Kama muta-related posts had higher probability of being engaged (i.e., likes, comments and sharing).	X	X

Note. The number of the particular study where each stimulus was used is referenced after the S; P included those that were referenced as Pilot studies in the published articles. Effects on behaviour are classified in terms of direct or indirect. That is, whether the main effects described would impact it directly (actual behaviour, such as amount of time devoted to help) or indirectly (tendency or motivation to, such as increases intention to help others), respectively.

CFA and Reliability Analyses

Supplemental Table S2 presents the summary of factorial structures of all scales used in the study.

Supplemental Table S2.

Fit and Reliability Indexes for each Scale Used in the Analyses.

Scales and dimensions	X^2	*df*	RMSEA	IC 95% RMSEA	CFI	TLI	SRMR	α	Ω
Awe total	190.889	73	.067	.058, .077	.975	.968	.050	.953	.966
Affective resp.	-	-	-	-	-	-	-	.946	.950
Physiological resp.	-	-	-	-	-	-	-	.870	.871
Cognitive-Subjective resp.	-	-	-	-	-	-	-	.859	.873
Action tendency	-	-	-	-	-	-	-	.862	.864
Help intention in NGOs (after scale)	23.135	2	.172	.120, .229	.952	.856	.042	.816	.827
Elevation total	395.855	147	.071	.064, .078	.949	.940	.058	.963	.979
Appraisals	-	-	-	-	-	-	-	.920	.922
Affective resp.	-	-	-	-	-	-	-	.948	.950
Physiological resp.	-	-	-	-	-	-	-	.904	.908
Cognitive-Subjective resp.	-	-	-	-	-	-	-	.903	.906
Action tendency	-	-	-	-	-	-	-	.959	.959
Help intention in NGOs (after scale)	11.004	2	.115	.070, .166	.966	.898	.035	.813	.820
Kama Muta total	551.484	222	.064	.058, .069	.927	.916	.067	.946	.968
Tears	-	-	-	-	-	-	-	.867	.871
Chills	-	-	-	-	-	-	-	.887	.888
Warmth	-	-	-	-	-	-	-	.876	.876
Talk	-	-	-	-	-	-	-	.828	.831
Enthusiasm	-	-	-	-	-	-	-	.615	.634
Appraisals	-	-	-	-	-	-	-	.914	.914
Motivations	-	-	-	-	-	-	-	.911	.912
Labels	-	-	-	-	-	-	-	.836	.850
Help intention in NGOs (after scale)	116.220	2	.139	.092, .192	.963	.888	.039	.830	.836
Transcendence values	64.674	5	.106	.090, .123	.929	.858	.047	.750	.758
Wellbeing								$r_{(1063)}$ = .668	

Note: Fit indexes were Chi-square (X^2), df, degrees of freedom; RMSEA, Root Mean Square Error of Approximation; CFI, Comparative Fit Index; TLI, Tucker-Lewis Index; SRMR, Standardized Root Mean Square Residual; α, Chronbach's alpha; Ω, McDonald's Omega. Due to Wellbeing was measured by two items, we report here the Pearson bivariate correlation (p < .001).

SEM Analyses

Supplemental Figures S1-S3 present a visual representation of all SEM models, as well as the information of the models' fit.

Supplemental Figure S1.

SEM Analyses of the Effects of Awe on Fusion of Identity, Helping Intention and Wellbeing.

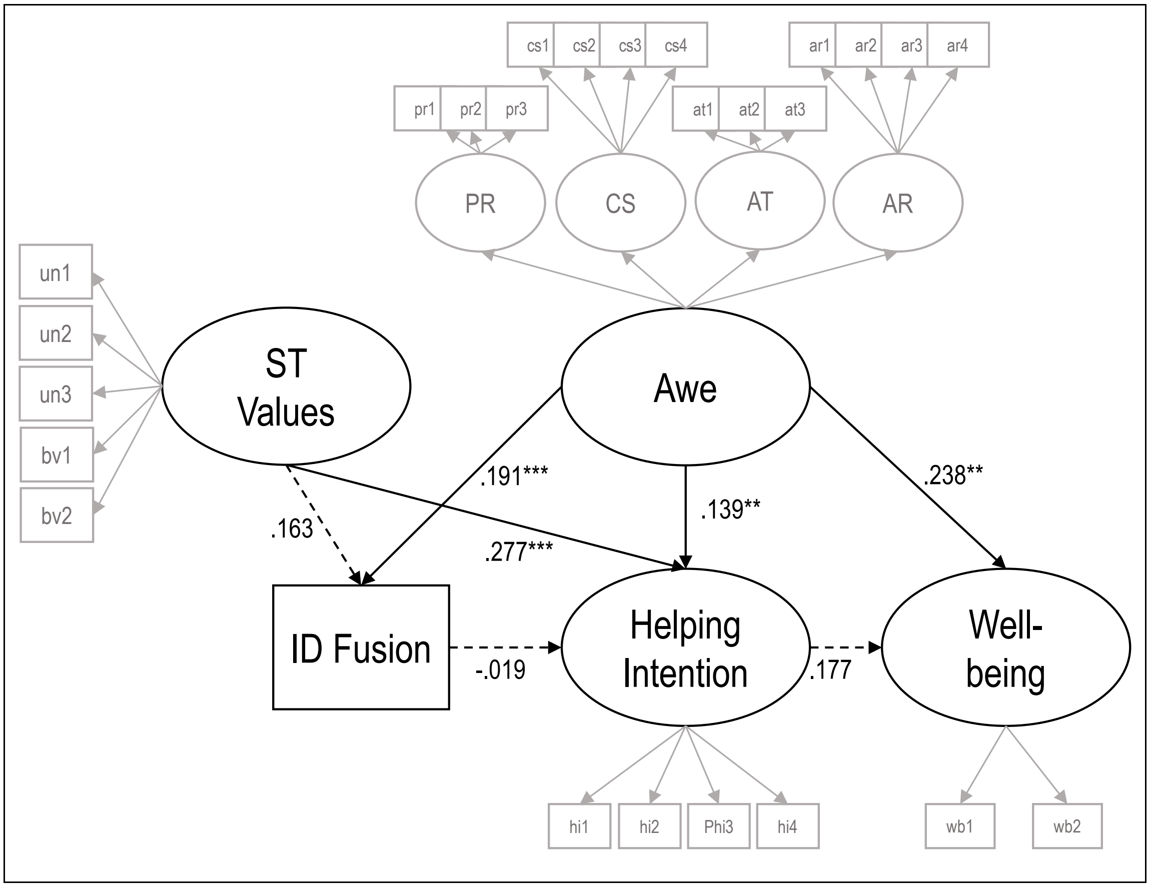

Note. Dashed lines show significant relationships †, *, **, *** indicate *p* values of < .10, .05, .01, and .001, respectively. Model's fit: $X^2_{(288)}$ = 627.77, RMSEA = .057, IC90% [.052, .063], CFI = .935, TLI = .927, SRMR = .064.

Supplemental Figure S2.

SEM Analyses of the Effects of Elevation on Fusion of Identity, Helping Intention and Wellbeing.

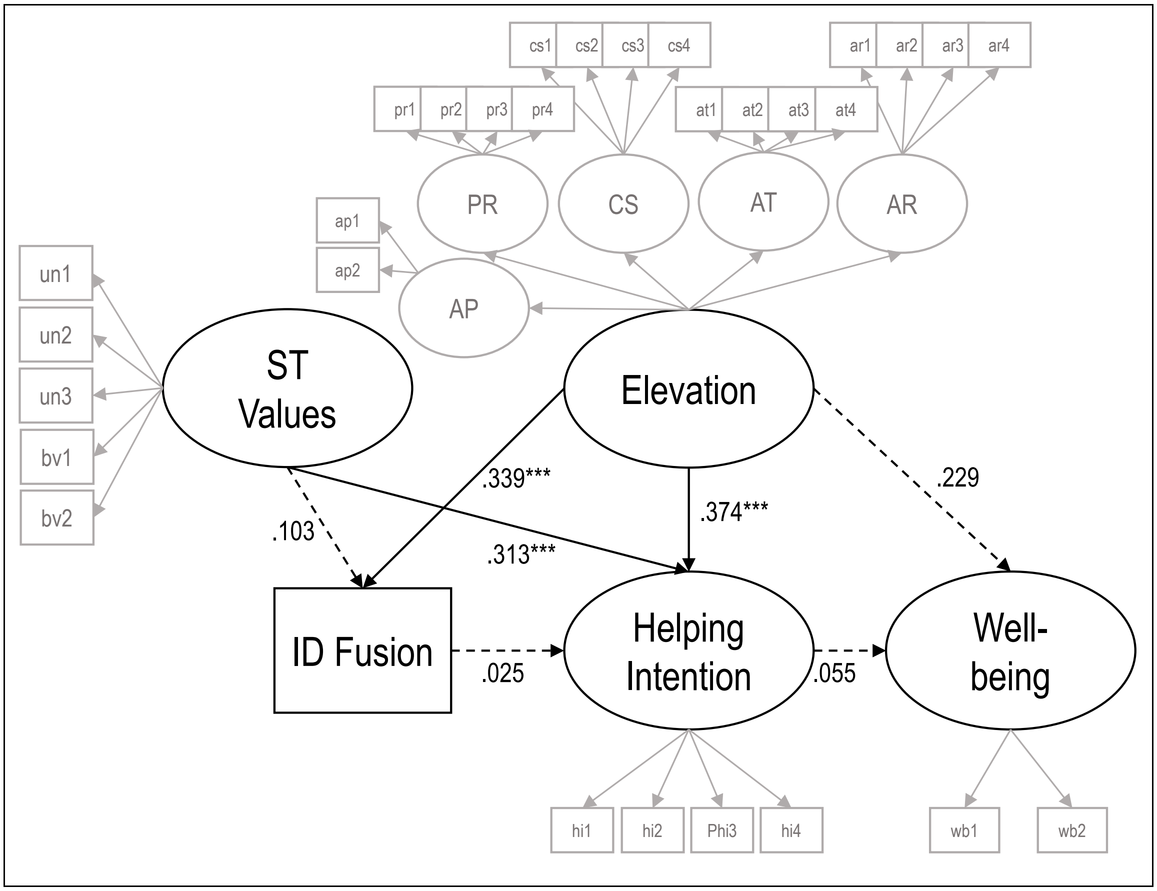

Note. Dashed lines show significant relationships †, *, **, *** indicate *p* values of < .10, .05, .01, and .001, respectively. Model's fit: $X^2_{(422)}$ = 903.92, RMSEA = .058, IC90% [.053, .063], CFI = .920, TLI = .911, SRMR = .058.

Supplemental Figure S3.

SEM Analyses of the Effects of Kama Muta on Fusion of Identity, Helping Intention and Wellbeing.

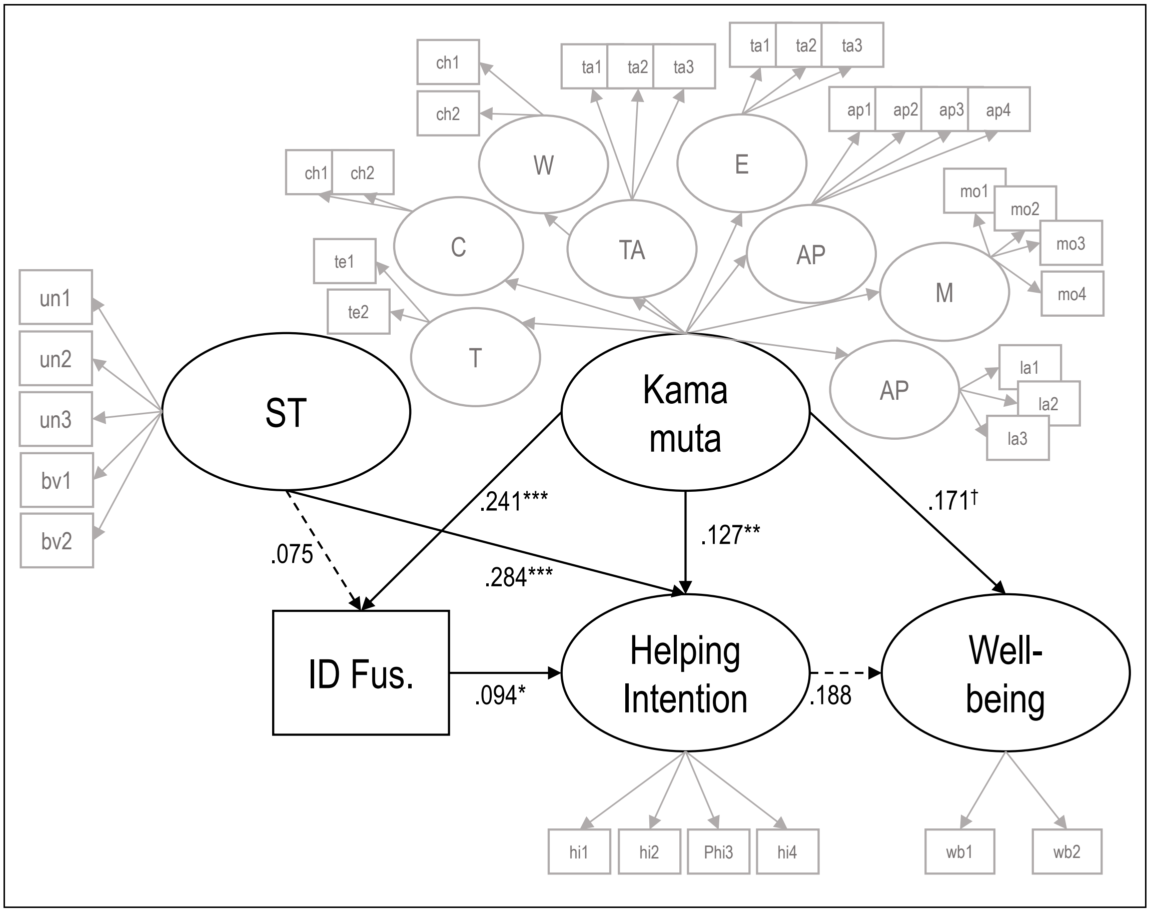

Note. Dashed lines show significant relationships †, *, **, *** indicate *p* values of < .10, .05, .01, and .001, respectively. Model's fit: $X^2_{(545)} = 1123.48$, RMSEA = .054, IC90% [.050, .058], CFI = .918, TLI = .911, SRMR = .062.

Random Model Meta-Analyses across Samples

Supplemental Figures S4-S6 show forest plots of the random model meta-analyses of STEs and dependent variables. These relationships were conducted across samples, being from UPV/EHU (The Basque Country, n = 112), PUCE (Ecuador, n = 256), and UNED (Spain [whole country], n = 695).

Supplemental Figure S4.

Random Model Meta-Analyses of the Relationship of Awe and Dependent Variables across Samples.

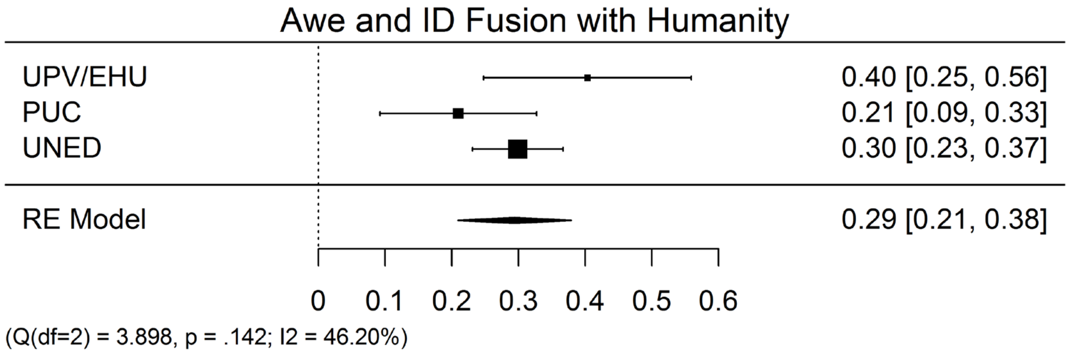

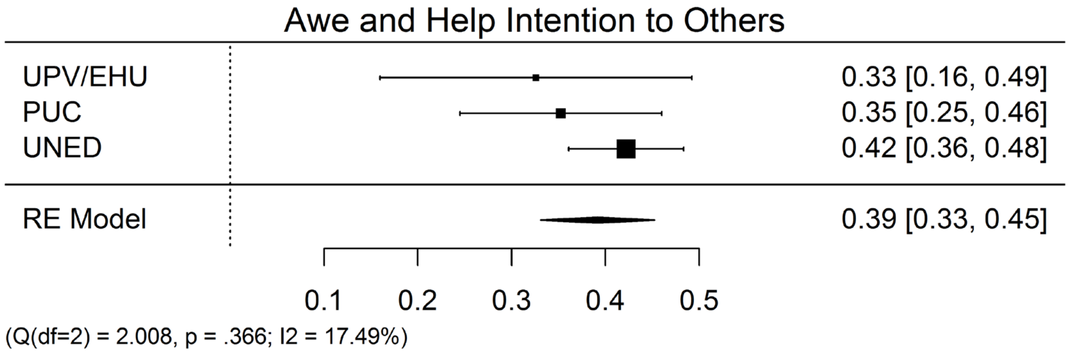

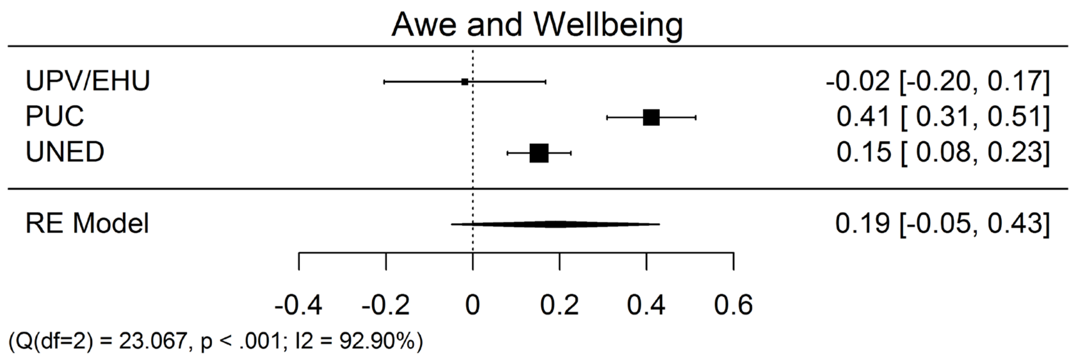

Note. Random effects meta-analysis of the correlation between Awe (total) and Identity Fusion with Humanity (top), Help Intention to Others (center), and Wellbeing (bottom). Effect size is Pearson's r [95% CI]. Q indicates Q-tests of heterogeneity; I2 indicates I^2.

Supplemental Figure S5.

Random Model Meta-Analyses of the Relationship of Elevation and Dependent Variables across Samples.

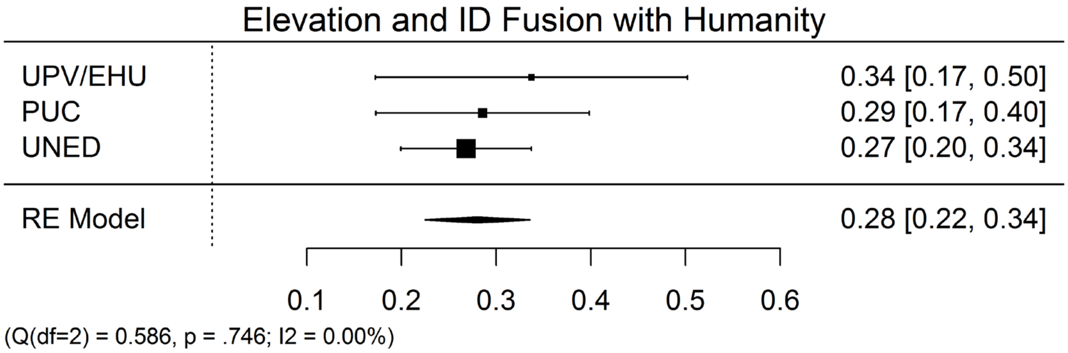

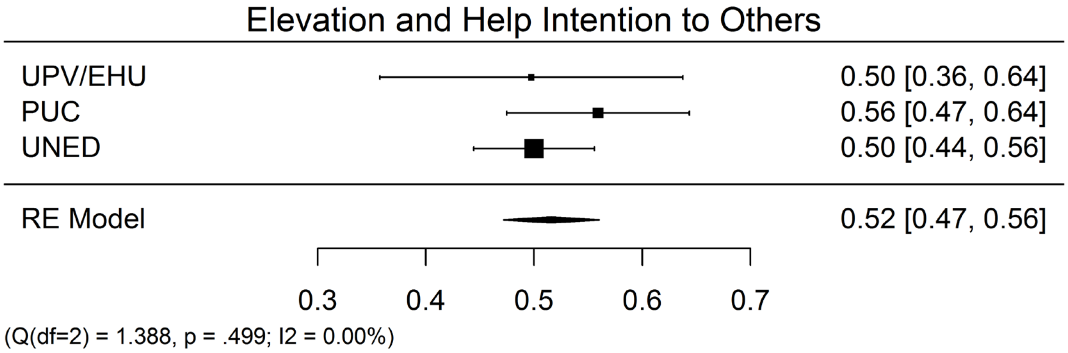

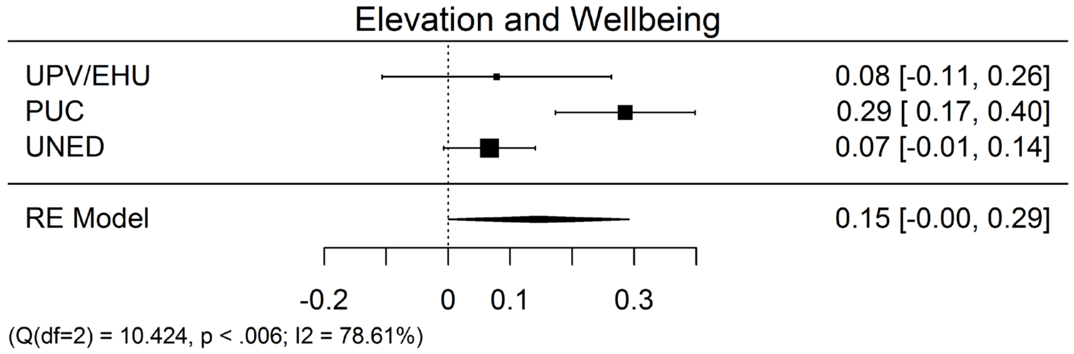

Note. Random effects meta-analysis of the correlation between Elevation (total) and Identity Fusion with Humanity (top), Help Intention to Others (center), and Wellbeing (bottom). Effect size is Pearson's *r* [95% CI]. Q indicates *Q*-tests of heterogeneity; I2 indicates I^2.

Supplemental Figure S6.

Random Model Meta-Analyses of the Relationship of Kama Muta and Dependent Variables across Samples.

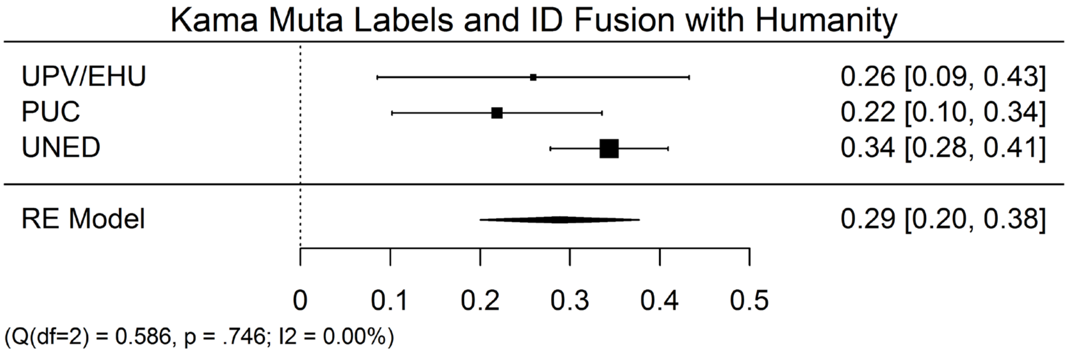

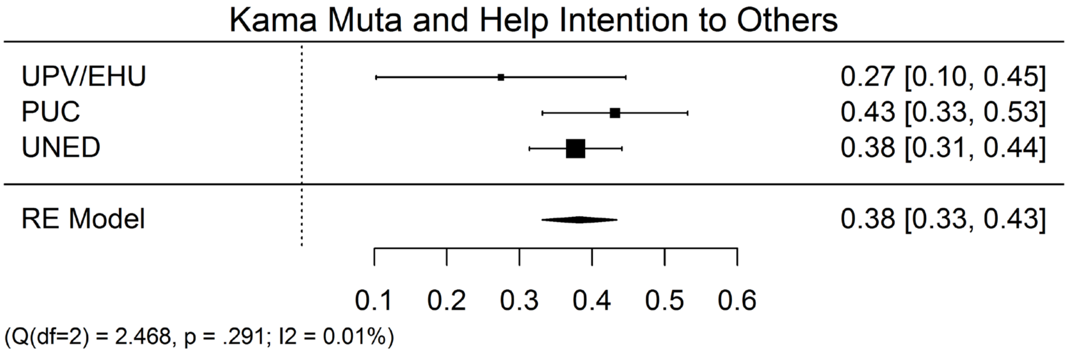

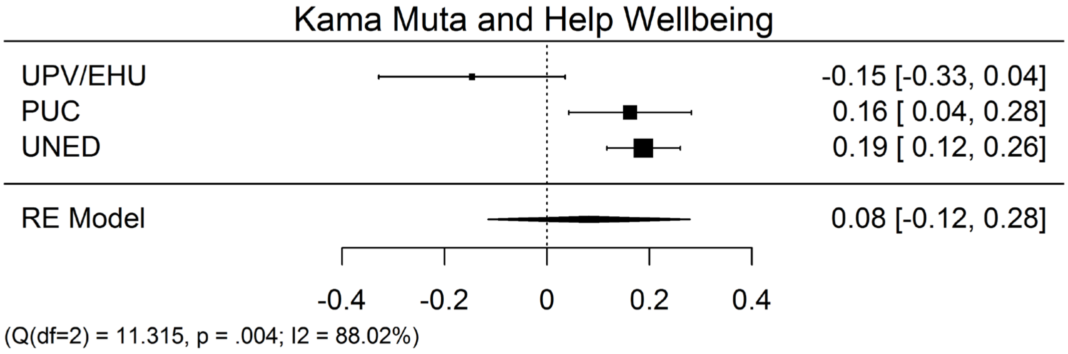

Note. Random effects meta-analysis of the correlation between Kama Muta (total) and Identity Fusion with Humanity (top), Help Intention to Others (center), and Wellbeing (bottom). Effect size is Pearson's *r* [95% CI]. Q indicates *Q*-tests of heterogeneity; I2 indicates I^2.
